# Twist1 regulates macrophage plasticity to promote renal fibrosis through galectin-3

**DOI:** 10.1007/s00018-022-04137-0

**Published:** 2022-02-19

**Authors:** Qingfeng Wu, Shiren Sun, Lei Wei, Minna Liu, Hao Liu, Ting Liu, Ying Zhou, Qing Jia, Di Wang, Zhen Yang, Menglu Duan, Xiaoxia Yang, Peisong Gao, Xiaoxuan Ning

**Affiliations:** 1 Department of Geriatrics, Xijing Hospital, Fourth Military Medical University, No. 127 Chang le West Road, Xi’an, 710032 Shaanxi China; 2 Department of Geriatrics, Ninth Hospital of Xi’an City, Xi’an, 710054 Shaanxi China; 3 Department of Nephrology, Xijing Hospital, Fourth Military Medical University, Xi’an, 710032 Shaanxi China; 4 State Key Laboratory of Cancer Biology, Fourth Military Medical University, Xi’an, 710032 Shaanxi China; 5 Division of Allergy and Clinical Immunology, Johns Hopkins University School of Medicine, Johns Hopkins Asthma and Allergy Center, 5501 Hopkins Bayview Circle, Room 2B. 71B, Baltimore, MD 21224 USA; 6 Johns Hopkins Asthma and Allergy Center, 5501 Hopkins Bayview Circle, Room 3B.71, Baltimore, MD 21224 USA

**Keywords:** Twist1, Macrophage, Polarization, Galectin-3, Renal fibrosis

## Abstract

Renal interstitial fibrosis is the pathological basis of end-stage renal disease, in which the heterogeneity of macrophages in renal microenvironment plays an important role. However, the molecular mechanisms of macrophage plasticity during renal fibrosis progression remain unclear. In this study, we found for the first time that increased expression of Twist1 in macrophages was significantly associated with the severity of renal fibrosis in IgA nephropathy patients and mice with unilateral ureteral obstruction (UUO). Ablation of Twist1 in macrophages markedly alleviated renal tubular injury and renal fibrosis in UUO mice, accompanied by a lower extent of macrophage infiltration and M2 polarization in the kidney. The knockdown of Twist1 inhibited the chemotaxis and migration of macrophages, at least partially, through the CCL2/CCR2 axis. Twist1 downregulation inhibited M2 macrophage polarization and reduced the secretion of the profibrotic factors Arg-1, MR (CD206), IL-10, and TGF-β. Galectin-3 was decreased in the macrophages of the conditional Twist1-deficient mice, and Twist1 was shown to directly activate galectin-3 transcription. Up-regulation of galectin-3 recovered Twist1-mediated M2 macrophage polarization. In conclusion, Twist1/galectin-3 signaling regulates macrophage plasticity (M2 phenotype) and promotes renal fibrosis. This study could suggest new strategies for delaying kidney fibrosis in patients with chronic kidney disease.

**Supplementary Information:**

The online version contains supplementary material available at 10.1007/s00018-022-04137-0.

## Introduction

Chronic kidney disease (CKD) is characterized by excessive extracellular matrix deposition and chronic inflammation and is highly prevalent worldwide [1]. The prevalence of CKD in adults in the United States is about 13%, while in China, the prevalence of CKD is about 12% [2]. Renal fibrosis, including tubulointerstitial fibrosis, tubular atrophy, and glomerulosclerosis, is the common pathogenesis for all CKD [3], and is an integral part of the progression to end-stage renal disease [4]. Current treatment options for CKD are limited and there is an urgent need for new therapeutic targets.

Recent research elucidated the role of macrophage plasticity and functional heterogeneity during the progression from kidney inflammation to renal fibrosis [5, 6]. In response to tissue insults, tissue-infiltrating as well as resident macrophages undergo phenotypic transition and display functional diversity in their adaptation to the local microenvironment [7]. These macrophages differentiate into proinflammatory classically-activated phenotypes (M1) or into wound healing/profibrotic alternatively activated (M2) phenotypes [8]. Both macrophage phenotypes promote kidney fibrosis [9]. M1 macrophages can cause local tissue damage by inducing the apoptosis of the surrounding cells and releasing proinflammatory substances such as tumor necrosis factor (TNF)-α and large amounts of nitric oxide (NO) through the inducible NO synthase (iNOS) [10, 11]. These proinflammatory factors promote fibrosis through inhibited degradation of fibrinogen by the inhibition of matrix metalloproteinases [12]. Using a different pathway, M2 macrophages can produce a large amounts of tumor growth factor-beta (TGF-β), vascular endothelial growth factor (VEGF), and Type IV collagen α (Col4α), that promote the secretion of profibrotic factors as well as the differentiation of fibroblasts [13]. Excessive extracellular matrix and profibrotic growth factors resulting from accumulated M1/M2 macrophages promotes the development of renal fibrosis [14]. Nevertheless, the mechanisms that drive macrophage chemotaxis, polarization, and mediation of collagen production in the kidney remain unclear.

**Fig. 1:**
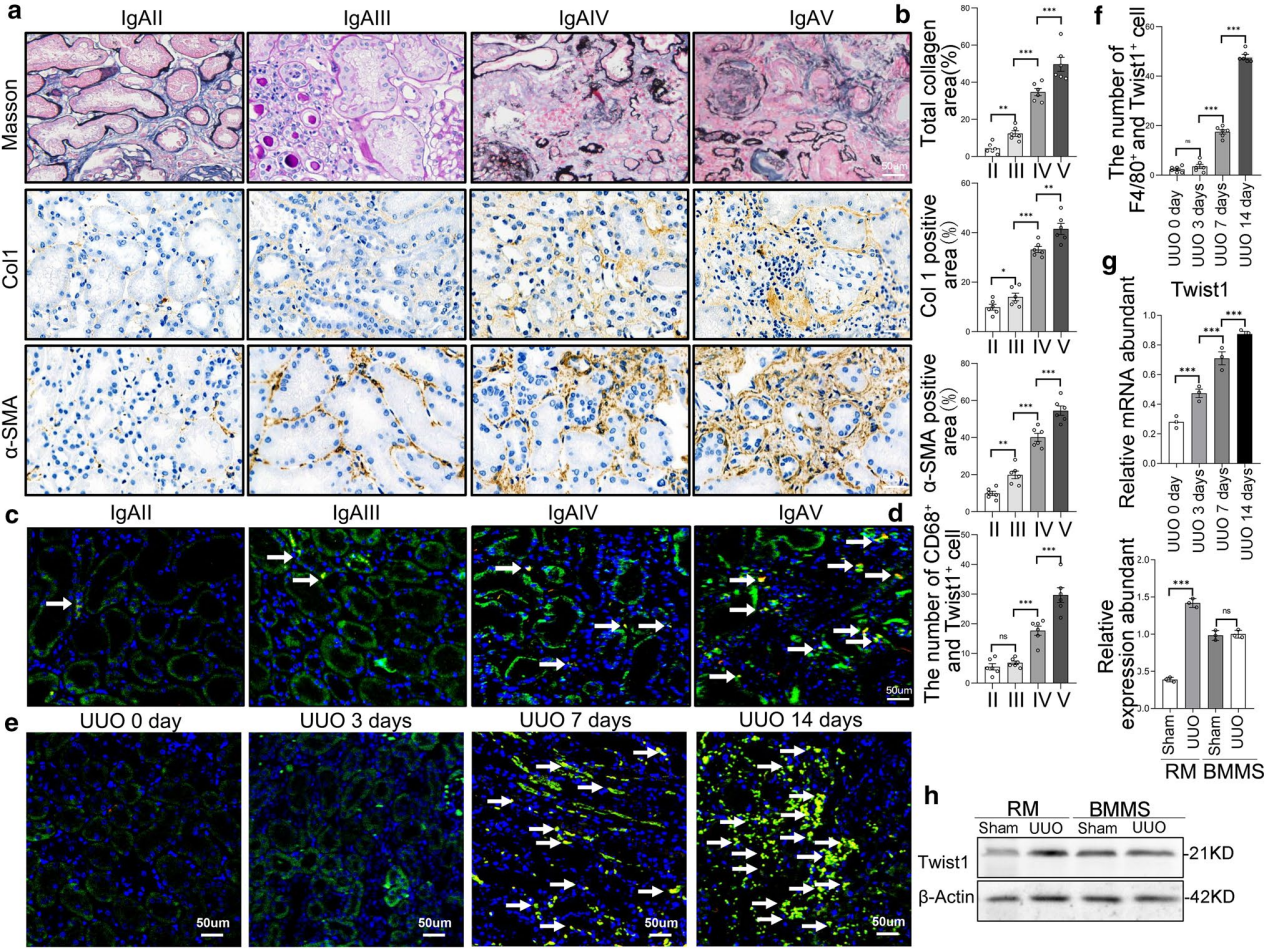
Twist1 signaling is activated in macrophages from fibrotic kidneys. **a** Representative photos of renal sections from biopsy samples from patients with IgAN. HE, Masson, Col-1, and a-SMA immune staining (× 20) of 3-μm kidney sections for fibrosis area. Bar scale = 50 μm, *n* = 6 samples per group, *n* = 10 micrographs analyzed per mouse. **b** Bar graph analysis of the fibrotic area, total collagen content, Col-1, and α-SMA staining positive area in IgAN patient sections. **c** Representative immunofluorescence (× 40) for Twist1 (green), CD68 (red; macrophage), and DAPI (blue) in 3 μm kidney sections and analysis demonstrating Twist1 expression in macrophage of IgAN patients among groups. **d** Bar graph analysis of demonstrating Twist1 expression in macrophage of IgAN patients. **e** The mice were euthanized at 0, 3, 7, and 14 days after UUO, immunofluorescence (× 20) for Twist1 (green), F4/80 (red; macrophage), and DAPI (blue) in 3-μm kidney sections and analysis demonstrating Twist1 expression in macrophage of mouse mold after UUO. Bar scale = 50 μm, *n* = 6 samples per group, *n* = 10 micrographs analyzed per mouse. **f** Bar graph analysis of demonstrating Twist1 expression in macrophage of UUO 14 days mouse among groups. **g** Real-time PCR analysis of the mRNA abundance for Twist1 of renal macrophage sorted from mouse kidney after UUO 0, 3, 7, 14 days. *n* = 3 mice per group, *n* = 3 independent experiments. **h** Western blotting of Twist1 expression in BMMs and renal macrophage sorted from mice at 14 days after UUO. *n* = 6 mice per group, *n* = 3 independent experiments. **i** Bar graph analysis of Twist1 expression in RM and BMMs. *P < 0.05, **P < 0.01. Data are presented as the mean ± SEM. Data were first analyzed for normal distribution, and if data passed normality test, two-tailed Student’s *t* test for two groups and two-way ANOVA for multiple groups was used. *IgAN* IgA nephropathy, *Col-1* collagen type 1, *α-SMA* alpha-smooth muscle actin, *BMMs* bone marrow-derived macrophage, *RM* renal macrophage

The initiation and resolution of require mechanisms for the comprehensive reprogramming of macrophage interactions with epithelial cells and fibroblasts [15]. These mechanisms involve several transcription factors, including nuclear factor (NF)-κB [16], interferon regulatory factors (IRFs) [17], signal transducers and activators of transcription (STATs) [18], wingless-INT (Wnt) [19], and activator protein 1 (AP-1) [20]. Twist1, a member of the basic helix–loop–helix family of transcription factors, has multiple functions that are associated with fibrotic diseases and tumor progression [21]. Plenty of evidences showed Twist1 promoted fibrosis diseases including skin fibrosis [22], pulmonary fibrosis [23], and liver fibrosis [24]. We and others have demonstrated that Twist1 expression was elevated in renal tubular epithelial cells and is involved in an epithelial mesenchymal transition (EMT) program implicated in renal fibrosis [25, 26]. In fact, it is partial EMT that plays essential roles in renal fibrosis [27]. To further explored the mechanism of Twist1 in kidney fibrosis, we made the unexpected discovery that Twist1 is also highly expressed in renal macrophages in the unilateral ureteral obstruction (UUO) mouse model (Fig. 1e). This led us to investigate whether transcription factor Twist1 expressed by renal macrophages may regulate macrophage plasticity and heterogeneity to promote renal fibrosis.

In this study, we characterized the effect of Twist1 on macrophage polarization and on the progression of renal fibrosis and investigated the underlying mechanisms. In vitro and in vivo studies revealed that Twist1-regulated macrophage chemotaxis and M2-type polarization that promoted renal fibrogenesis through a positive regulation galectin-3. This study suggests that inhibiting Twist1 or its target galectin-3 could be a potential new therapeutic strategy to prevent or treat renal fibrosis.

### Results

### Twist1 expression is increased in macrophages of fibrotic renal disease

Increasing evidence suggests that kidney fibrosis requires the establishment of a regulated inflammatory response mediated by infiltrating monocyte/macrophages [28]. Although the Twist1 has been well studied in kidney fibrosis by regulating renal tubular epithelial cell and migration of tumor cell [29], little is known about the Twist1 in regulation of renal monocyte/macrophage function in normal and chronic kidney disease. It is well established that in inflammatory disease states, such as in IgA nephropathy (IgAN) [30] and mice model with unilateral ureteral obstruction (UUO), monocyte/macrophages remain persistently in an inflammatory response and fail to transition phenotype to protective state [31]. To determine if the Twist1 is altered in inflammatory monocytes from patients with IgAN, we investigated biopsies from IgAN patients with Lee's grade I–V and a glomerular filtration rate (GFR) of 30–125 ml/min (Supplementary Information Table 1). The severity of renal interstitial fibrosis was determined by immunohistochemistry of alpha-smooth muscle actin (α-SMA) and collagen1 (Col-1) (Fig. 1a, b). The expression of Twist1 in macrophages was increased in renal specimens from patients with IgAN IV–V compared with those with IgAN II–III (Fig. 1c, d), possibly inferring an association between Twist1 expression and advanced renal fibrosis. These findings were supported by analyzing Twist1 expression in macrophages in the kidneys from the mouse UUO model (Supplementary Information Fig. 1a, b). Expression of Twist1 by renal macrophages in renal interstitium gradually increased on days 3, 7, and 14 after UUO, consistent with the collagen area in the renal interstitium (Fig. 1e, f), which was further validated by RT-PCR (Fig. 1g). This increased expression of Twist1 in macrophage of renal tissue is highly relevant as monocytes are recruited from the blood that transition to macrophages after renal injury. Next, to analyze role of Twist1 in macrophage of renal tissue or marrows in kidney fibrosis, renal macrophages (RM) and bone marrow macrophages (BMMs) were isolated from mice model with and without UUO. Increased expression of Twist1 was observed in macrophage sorted from kidney of mice but not in BMMs after UUO treatment, as observed by western blotting (Fig. 1h, i).

### Ablation of Twist1 in macrophages ameliorates renal fibrosis in UUO mice

To explore the role of Twist1 in macrophage activation in kidney fibrosis, we created mice with Twist1-deficient myeloid cells. Twist1 floxed mice were mated with Cre mice controlled by mouse myeloid cell-specific lyz2 promoter to obtain *Lyz2-Cre* + *Twist1fl/fl* mice (Supplementary Information Fig. 2a). The same gender with genotyping *Lyz2-Cre-Twist1fl/fl* littermates were referred to as wild-type (WT) or control mice. A representation of the mouse Twist1 wild-type allele and the targeted allele is shown in Supplementary Fig. 2b. To confirm that Twist was ablated in macrophages, western blot (Supplementary Information Fig. 2c) and RT-PCR (Supplementary Information Fig. 2d) were performed. Twist1 expression was reduced in mice compared with control *Lyz2-Cre-Twist1fl/fl* littermates. Similarly, reduced expression of Twist1 in the renal macrophages of *Lyz2-Cre* + *Twist1fl/fl* mice was detected using co-immunofluorescence staining with F4/80, a macrophage-specific marker (Supplementary Information Fig. 2e, f). In addition, at 8 weeks of age, *Lyz2-Cre* + *Twist1fl/fl* mice demonstrated no changes in body weight (Supplementary Information Fig. 2g), kidney-to-body weight ratio (Supplementary Information Fig. 2h), or renal tubular structure as detected by electron microscopy (Supplementary Information Fig. 2i) compared with control mice. Those data indicate that myeloid-specific Twist1-deficient mice were constructed successfully and that Twist1 ablation in macrophage did not affect mouse kidney and systemic development.

**Fig. 2:**
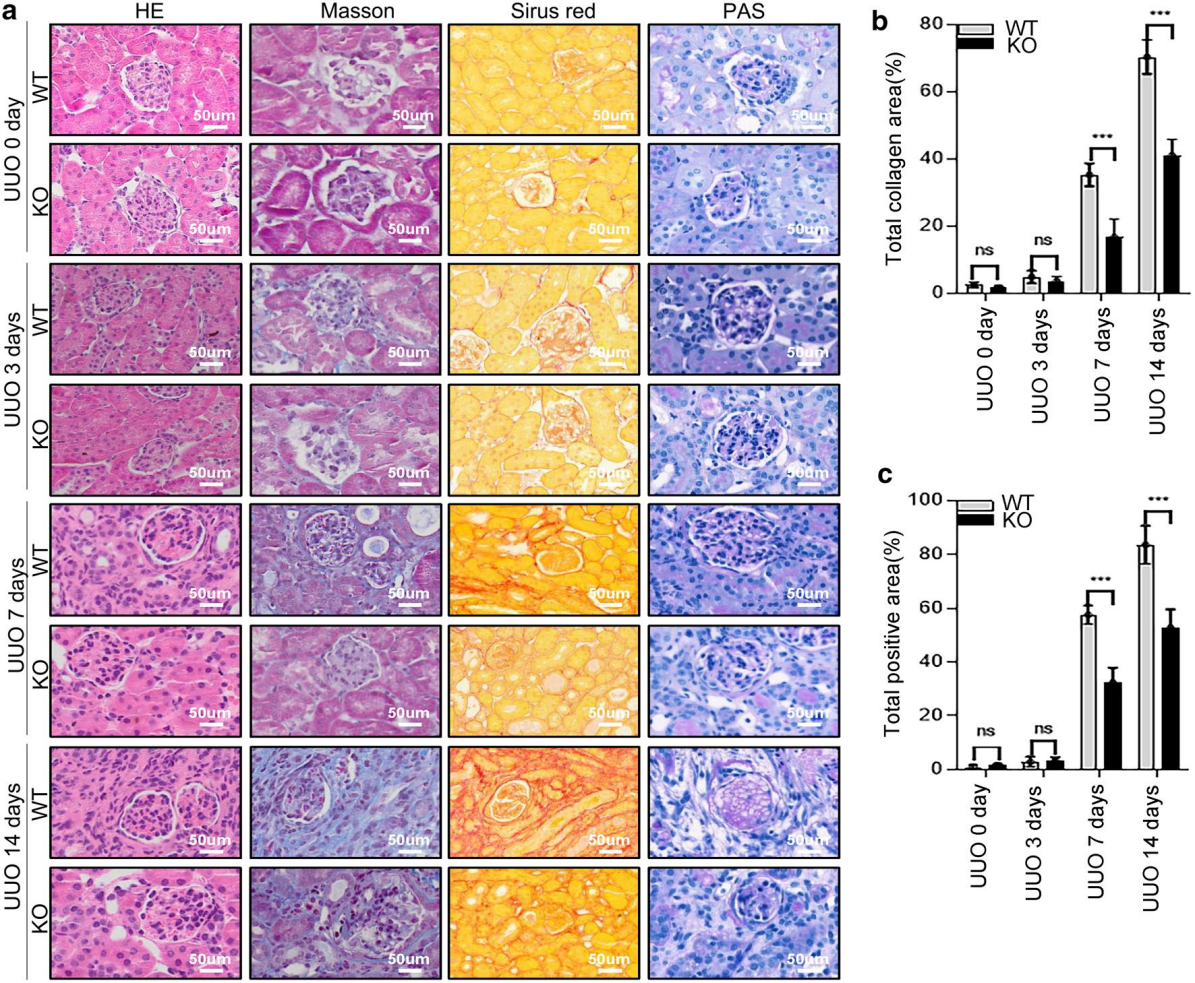
The deletion of Twist1 in macrophages improves interstitium fibrosis from injury in UUO mice. **a** HE, Masson, Sirus red, and PAS immune staining (× 20) of 3 μm kidney sections analysis fibrotic area, and total collagen content in UUO mice kidney tissues among groups as indicated. Bar scale = 50 μm, *n* = 5 animals per group, *n* = 10 micrographs analyzed per mouse. **b**,** c** Bar graph analysis of the fibrotic area, total collagen content (**b**, masson) and **c** collagen III and IV staining positive area (Sirus red) in UUO mice kidney tissues among groups as indicated. Bar scale = 50 μm, *n* = 5 animals per group, *n* = 10 micrographs analyzed per mouse. *P < 0.05, **P < 0.01. Data are presented as the mean ± SEM. Data were first analyzed for normal distribution, and if data passed normality test, two-tailed Student’s *t* test for two groups and two-way ANOVA for multiple groups was used

**Fig. 3:**
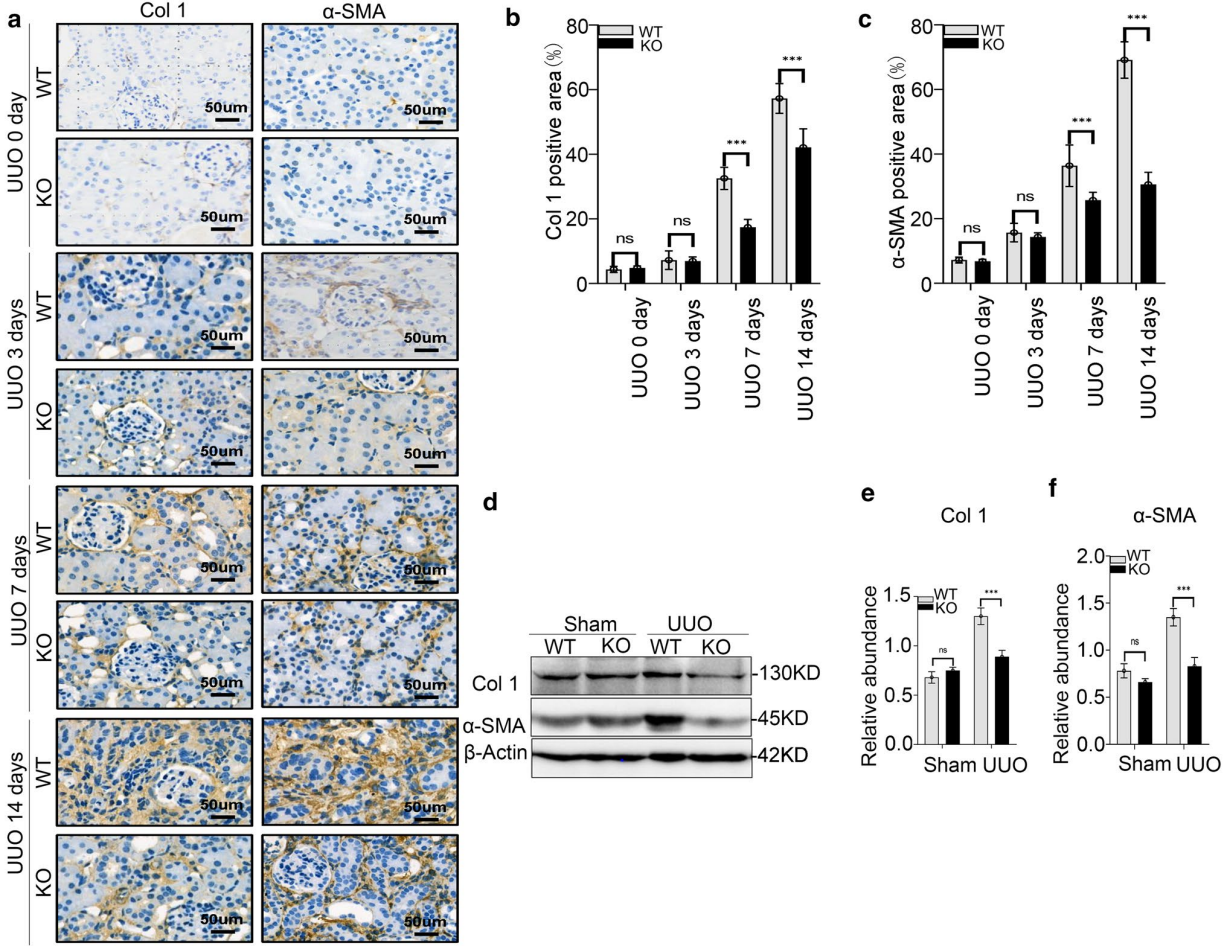
The ablation of Twist1 in macrophages reduces fibrotic factors in kidney after UUO mice. **a** Col-1 and α-SMA staining positive areas in UUO mice kidney tissues among groups as indicated. Bar scale = 50 μm, *n* = 5 animals per group, *n* = 10 micrographs analyzed per mouse. **b**, **c** Bar graph analysis of the Col-1 (**b**), and α-SMA (**c**) staining positive area in UUO mice kidney tissues among groups as indicated. **d** Western blotting analyses of α-SMA and Col-1 expression in renal tissue at 14 days after UUO. **e**, **f** Bar graph analysis of Col-1 (**e**) and α-SMA (**f**) relative expression in renal tissue at 14 days after UUO in *Cre*^+^*Twist1*^*fl/fl*^ and wild-type littermate mice. *P < 0.05, **P < 0.01. Data are presented as the mean ± SEM. Data were first analyzed for normal distribution, and if data passed normality test, two-tailed Student’s *t* test for two groups and two-way ANOVA for multiple groups was used

**Fig. 4:**
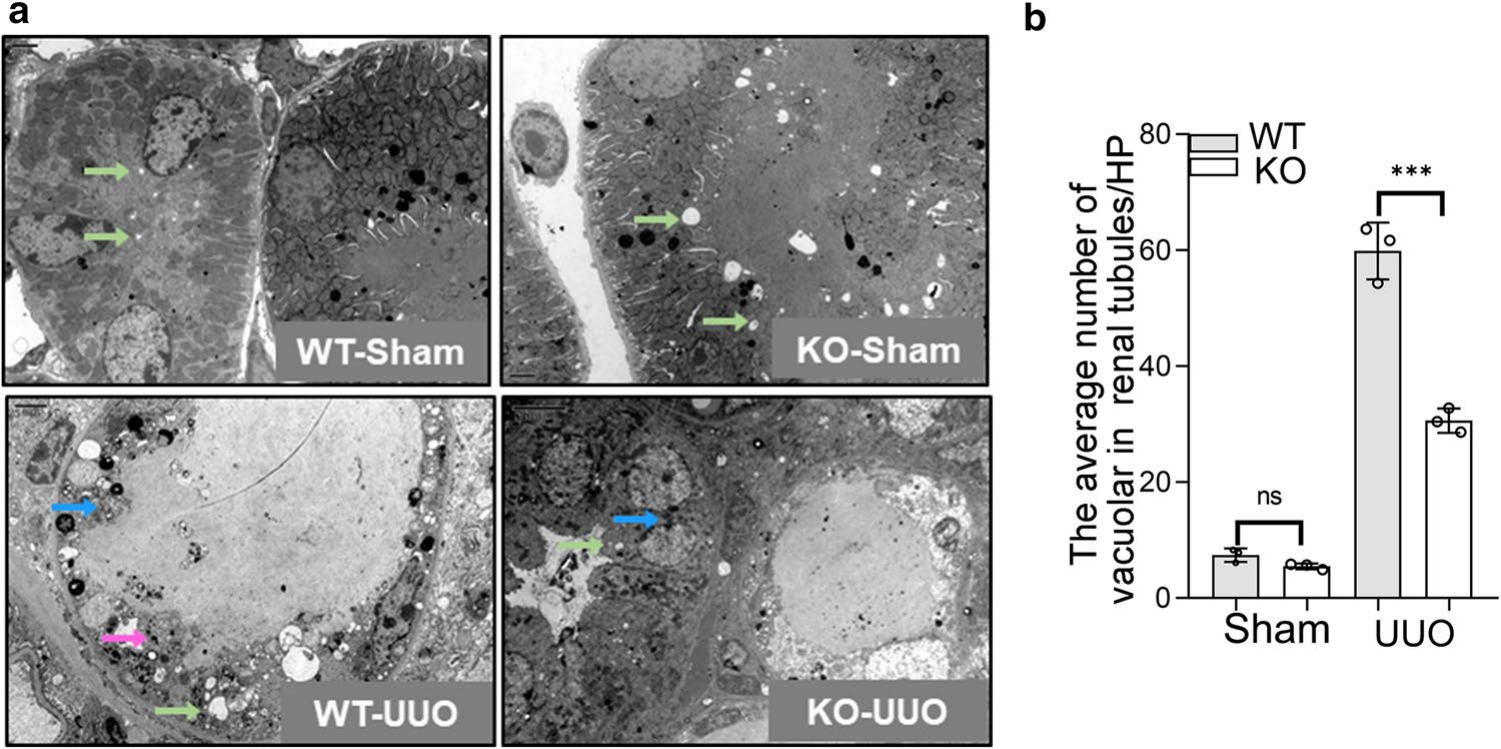
The ablation of Twist1 in macrophages protects renal tubules from injury in UUO mice. **a** Representative electron microscopy of renal tubular epithelial cells atrophy (magenta arrows), renal tubular epithelial cells fuse (blue allows), and mitochondrial vacuolar degeneration (green allows) in *Cre-Twist1fl/fl* and *Cre* + *Twist1fl/fl* mice after UUO. Bar scale = 2 μm. *n* = 3 animals per group, *n* = 3 micrographs analyzed per mouse. **b** Bar graph analysis of the mitochondrial vacuolar degeneration (a marker as renal tubules injury) in UUO mice kidney tissues among groups as indicated. *P < 0.05, **P < 0.01. Data are presented as the mean ± SEM. Data were first analyzed for normal distribution, and if data passed normality test, two-tailed Student’s *t* test for two groups and two-way ANOVA for multiple groups was used

We utilized transgenic mice to build UUO model mice. Histopathological analyses of fibrotic kidneys using H&E, Masson’s trichrome staining, Sirius red, and periodic acid–Schiff (PAS) staining showed improved tubular health and a lower degree of interstitial fibrosis in *Lyz2-Cre* + *Twist1fl/fl* mice (named as knockout mice, abbreviated to KO) compared with controls [littermates of the same gender with genotype *Lyz2-Cre-Twist1fl/fl* referred to as wild-type (WT)] on days 7 and 14 after UUO (Fig. 2a, b). Similarly, immunohistochemistry revealed that the expression of Col-1 and α-SMA, a common fibrotic factor, in the kidneys of the *Lyz2-Cre* + *Twist1fl/fl* mice was attenuated compared with WT mice (Fig. 3a–c). The reduced expression of Col-1 and α-SMA in the kidneys of the *Lyz2-Cre* + *Twist1fl/fl* mice was confirmed by western blot (Fig. 3d–f). In addition to the improvement of renal interstitial fibrosis, we further observed renal tubule structure by electron microscopy. There was less renal tubular mitochondrial vacuolation and tubular injury in the kidneys of the *Lyz2-Cre* + *Twist1fl/fl* mice than in the controls (Fig. 4a, b), which indicated that renal tubule structure is protected in Twist-deficiency in macrophage of mice. Overall, these results suggest that loss of Twist1 in macrophage relieved renal interstitial fibrosis and tubular injury.

### Twist1 deletion diminishes macrophage infiltration in UUO kidneys by regulating chemotaxis and migration

**Fig. 5:**
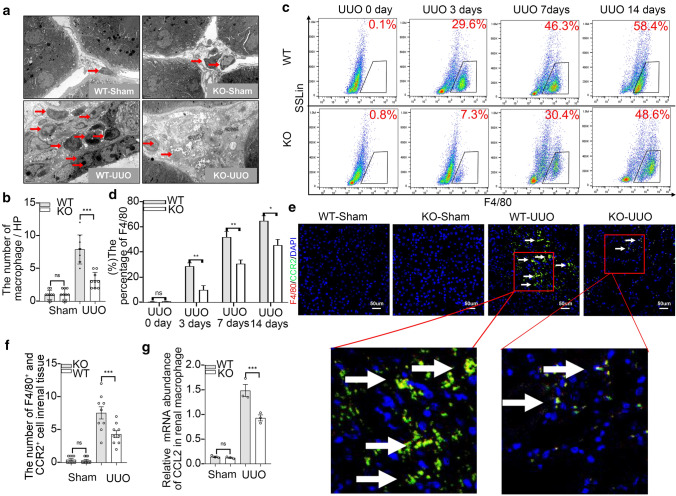
Twist1 ablation in macrophages reduces macrophage accumulation in UUO kidneys. **a** Representative electron microscopy and **b** bar graph analysis macrophage infiltration (red arrow) in *Cre* + *Twist1fl/fl* and wild-type littermate mice. Bar scale = 2 μm. *n* = 3 animals per group, *n* = 3 micrographs analyzed per mouse, *Cre* + *Twist1fl/fl* mice vs. wild-type littermate mice. **c** Flow cytometry and bar graph analysis (**d**) of macrophage infiltration in renal tissue *Cre* + *Twist1fl/fl* after UUO at 0, 3, 7, and 14 days versus macrophages from wild-type littermate UUO kidneys, *n* = 3 animals per group, *n* = 3 independent experiments. **e** Representative immunofluorescence (× 20) for CCR2 (Chemokine Receptors 2, white arrow) in 3 µm kidney sections and **f** bar graph analysis demonstrating CCR2 expression in all four groups of mice. Bar scale = 50 µm. *n* = 6 animals per group, *n* = 5 micrographs analyzed per mouse, WT-UUO vs. KO-UUO. **g** Real-time PCR analysis of the mRNA abundance for CCL2 (Chemokine ligand 2) in renal macrophage of *Cre* + *Twist1fl/fl* mice and wild-type littermates at 14 days after UUO (*n* = 3/group, repeated in triplicate). *P < 0.05, **P < 0.01. Data are presented as the mean ± SEM. Data were first analyzed for normal distribution, and if data passed normality test, two-tailed Student’s *t* test for two groups and two-way ANOVA for multiple groups was used

As we all know, macrophage plasticity plays essential function in immunity response including repair of injury and pathologic development [32]. Thus, we examined macrophage infiltration in UUO-induced injured kidney. Compared with the sham group, electron microscopy showed an increased macrophage accumulation in renal interstitium on day 14 after UUO in mice, but compared with WT mice, *Lyz2-Cre* + *Twist1fl/fl* mice showed reduced macrophage accumulation (Fig. 5a, b). Further flow cytometry assay revealed a time-dependent increase in F4/80^+^ macrophages from days 3 to 14 after UUO, however, less macrophage infiltration in *Lyz2-Cre* + *Twist1fl/fl* mice compared with WT mice with UUO after 3, 7, 14 days (Fig. 5c, d).

We next wanted to determine how did monocyte/macrophages move to injured renal tissue from bone morrow. Numerous reports suggested that macrophage chemotaxis is crucial during both onset and resolution of inflammation through the receptor–ligand CCR2–CCL2 (chemokine receptors 2–CHEMOKINE ligand 2) signaling axis [33]. Recent studies have found an increase in the expression of the CCR2 ligand, CCL2 (also called monocyte chemoattractant protein 1; MCP1) in the brain, kidney, liver after chronic hypoxia [34]. We examined the expression of CCR2 was reduced in the renal tissue of *Lyz2-Cre* + *Twist1fl/fl* mice on day 14 after UUO compared with control mice using immunofluorescence (Fig. 5e, f). Consistently, RT-PCR analysis revealed that the CCR2 ligand CCL2 was downregulated in renal macrophage from *Cre* + *Twist1fl/fl* mice (Fig. 5g). In vitro, small interfering RNA for Twist1 (siRNA-Twist1) was transfected into Raw264.7 cells and with empty vector transduction as a control. The silencing of Twist1 was confirmed by western blotting (Supplementary Information Fig. 3a). Using Transwell assays, we found a reduction in migration cells in Raw264.7 cells with siRNA-Twist1 compared with controls (Supplementary Information Fig. 3b, c). Intriguingly, the levels of CCL2 in the Transwell medium were reduced in Raw264.7 cells with siRNA-Twist1 (Supplementary Information Fig. 3d). These data suggest that the silencing of Twist1 in macrophages might reduce macrophage infiltration in UUO kidneys, at least partially, through the CCL2/CCR2 chemotaxis axis.

### Ablation of Twist1 in macrophages inhibits M2 macrophage polarization

**Fig. 6:**
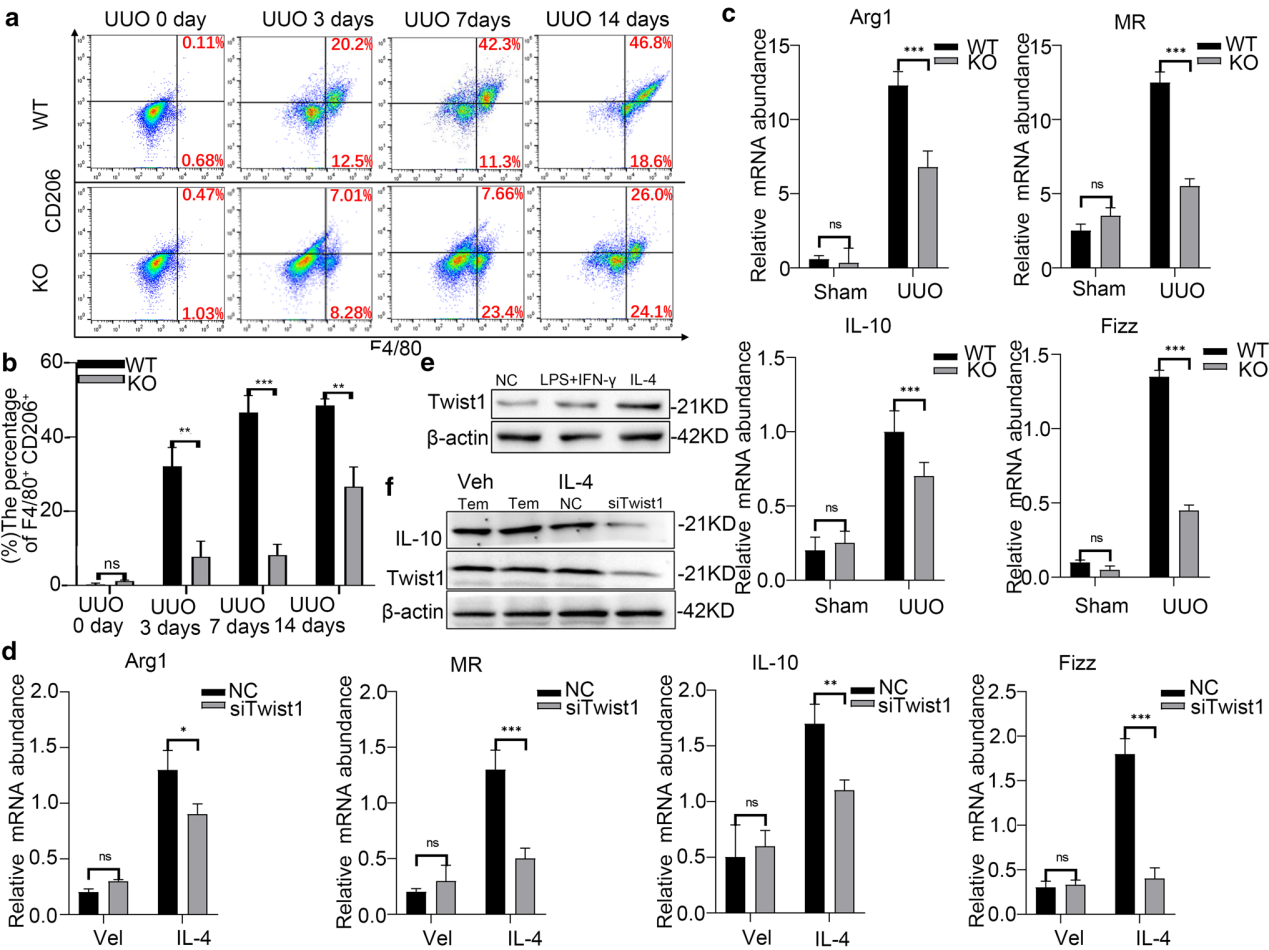
The ablation of Twist1 in macrophages diminishes M2 macrophage polarization in UUO kidneys. **a** Flow cytometry analysis and **b** bar graph analysis of M2 macrophage polarization in renal tissue of *Cre* + *Twist1fl/fl* and wild-type littermate mice at 0, 3, 7, and 14 days, *n* = 3 animals per group, *n* = 3 independent experiments, *Cre* + *Twist1fl/fl* and wild-type littermate mice. **c** Real-time PCR analysis showing the mRNA abundance for Arg-1, MR, IL10, and Fizz1 in macrophages from sham and UUO kidneys at 14 days after surgery. Each sample was pooled from five animals within the same group. Macrophages from *Cre* + *Twist1fl/fl* versus macrophages from wild-type littermate UUO kidneys, *n* = 3 independent experiments. **d** Western blotting for expression of Twist1 in Raw264.7 cells treated with PBS alone, IFN-γ plus LPS, IL-4, *n* = 3 independent experiments. **e** Western blot of Twist1 and IL-10 (M2 macrophage marker) expression in Raw264.7 cells after transfection with siRNA-Twist1 and empty vector controls, *n* = 3 independent experiments. **f** Real-time PCR analysis of the mRNA abundance for Arg-1, MR, IL-10, and Fizz1 in Twist1-silenced Raw264.7 cells, siRNA-Twist1 versus empty vector controls, *n* = 3 independent experiments. *P < 0.05, **P < 0.01. Data are presented as the mean ± SEM. Data were first analyzed for normal distribution, and if data passed normality test, two-tailed Student’s *t* test for two groups and two-way ANOVA for multiple groups was used

Macrophages differentiate into specific phenotypes in response to various microenvironmental stimuli and have specific biological functions [35]. To investigate the infiltrating macrophages phenotypes (M1/M2 subtype) in every assessment point of UUO model mice, flow cytometry assay of macrophages in fibrotic mouse kidneys revealed a time-dependent increase in the percentages of M2 macrophages (F4/80^+^CD206^+^) from days at 3 to 14 after UUO in WT littermates (Fig. 6a, b). Of note, the *Lyz2-Cre* + *Twist1fl/fl* mice showed smaller increases in the percentages of M2 macrophages compared with WT controls on days 3, 7, and 14 after UUO, but there were no significant differences between *Lyz2-Cre* + *Twist1fl/fl* and control mice in the proportions of classically activated M1 macrophages (F4/80^+^CD86^+^) at any assessment points after UUO (Supplementary Information Fig. 4a, b). RT-PCR revealed the same patterns in the mRNA expression of M2 macrophage-related genes (Arg-1, MR (CD206), IL-10, and Fizz1) in macrophages from fibrotic kidneys of *Lyz2-Cre* + *Twist1fl/fl* mice (Fig. 6c). By contrast, no significant differences were noted in the expression of M1 macrophage-related genes (TNF-α, IL-6, IL-1β, and iNOS) between *Lyz2-Cre* + *Twist1fl/fl* and control mice (Supplementary Information Fig. 4c). Western blot showed lower YM1 expression of the M2 marker in enriched macrophages from the kidneys of *Lyz2-Cre* + *Twist1fl/fl* mice on day 14 after UUO (Supplementary Information Fig. 4d).

In vitro, western blot showed upregulation of Twist1 expression in Raw264.7 cells stimulated with IL-4, but not in with IFN-γ plus LPS (Fig. 6d). We examined the role of Twist1 in macrophage polarization (M1 and M2) in Raw264.7 with or without Twist1 silencing (siRNA-Twist1), and then treated macrophages with IL-4 to induce M2 polarization (Fig. 6e). Similar to macrophages from fibrotic renal tissue in *Lyz2-Cre* + *Twist1fl/fl* mice, RT-PCR demonstrated that the silencing of Twist1 reduced IL-4-induced expression of the M2-associated genes Arg-1, MR, IL-10, and Fizz1 in macrophages (Fig. 6f). The effects of INF-γ plus LPS on iNOS, TNF-α, IL-6, and IL-12 were not significantly different between siRNA-Twist1 Raw264.7 and controls (Supplementary Information Fig. 4e). In brief, our data clearly illustrated that Twist1 in macrophage promoted macrophages towards anti-inflammatory M2 polarization.

### Ablation of Twist1 in macrophages inhibits the secretion of profibrotic growth factors or direct transition to myofibroblast-like cells

**Fig. 7:**
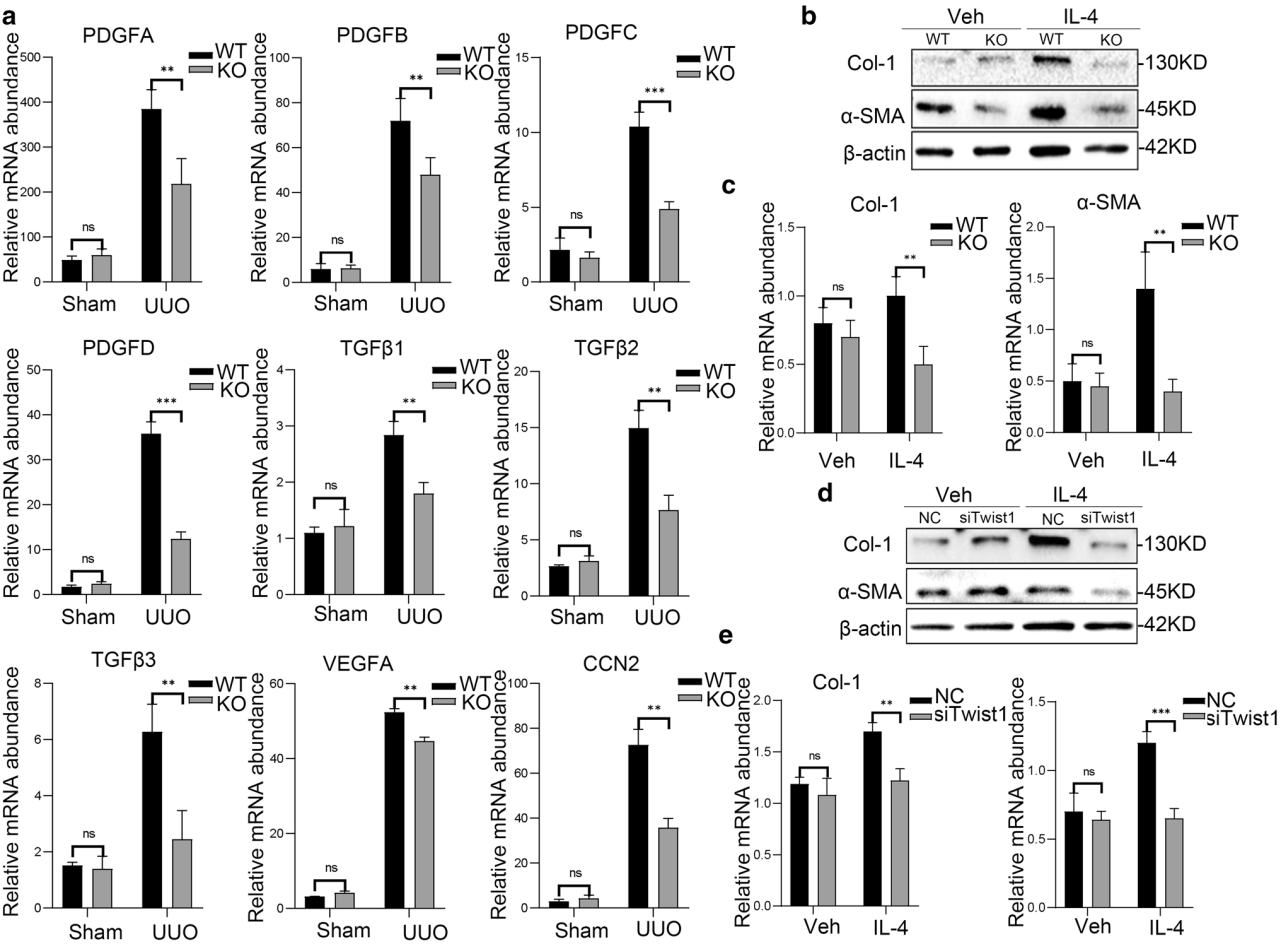
Twist1 deletion in macrophage inhibits section of the profibrotic growth factor. **a** Real-time PCR analysis showing the mRNA abundance for PDGFA, PDGFB, PDGFC, PDGFD, TGFβ1T, TGFβ2, TGFβ3, VEGFA, and CCN2 in macrophage sorted from fibrotic kidney at 14 days after surgery. Each sample was pooled from five animals within the same group, versus macrophages from wild-type littermates, *n* = 3 independent experiments. **b** Western blot of α-SMA and Col-1 in BMMs from Twist1-ablated mice and wild-type littermates stimulated with IL-4, *n* = 3 independent experiments. **c** Bar graph analysis α-SMA and Col-1 relative expression in BMMs from *Cre* + *Twist1fl/fl* mice and wild-type littermates stimulated with IL-4, *Cre* + *Twist1fl/fl* mice versus wild-type littermates. **d** Western blot of α-SMA and Col-1 in Twist1-silenced Raw264.7 and control stimulated with IL-4, *n* = 3 independent experiments. **e** Bar graph analysis of α-SMA and Col-1 relative expression in Twist1-silenced Raw264.7, and controls stimulated with IL-4, siRNA-Twist1 versus empty vector controls, *n* = 3 independent experiments. *P < 0.05, **P < 0.01. Data are presented as the mean ± SEM. Data were first analyzed for normal distribution, and if data passed normality test, two-tailed Student’s *t* test for two groups and two-way ANOVA for multiple groups was used

To identified that the roles of macrophage in kidney fibrosis, RT-PCR demonstrated that profibrotic cytokines of PDGFA, PDGFB, PDGFC, PDGFD, VEGFC, TGFb1, TGFb2, TGFb3, and CTGF secreted by M2 macrophages were upregulated in enriched macrophages from the fibrotic kidneys of both *Lyz2-Cre* + *Twist1fl/fl* mice and control littermates on day 14 after UUO (Fig. 7a), but their expression levels were lower in *Lyz2-Cre* + *Twist1fl/fl* mice compared with control mice. Western blot showed that the levels of fibrotic proteins, including Col-1 and α-SMA were lower in BMMs from fibrotic kidneys of the *Lyz2-Cre* + *Twist1fl/fl* mice compared with controls (Fig. 7b, c). This finding was supported by Twist1 silencing in Raw264.7 cells (Fig. 7d, e). In summary, these results indicate that Twist1 in macrophage may regulate macrophage transition to myofibroblast-like cells to promote renal fibrosis.

### Galectin-3 is a direct target of Twist1

**Fig. 8:**
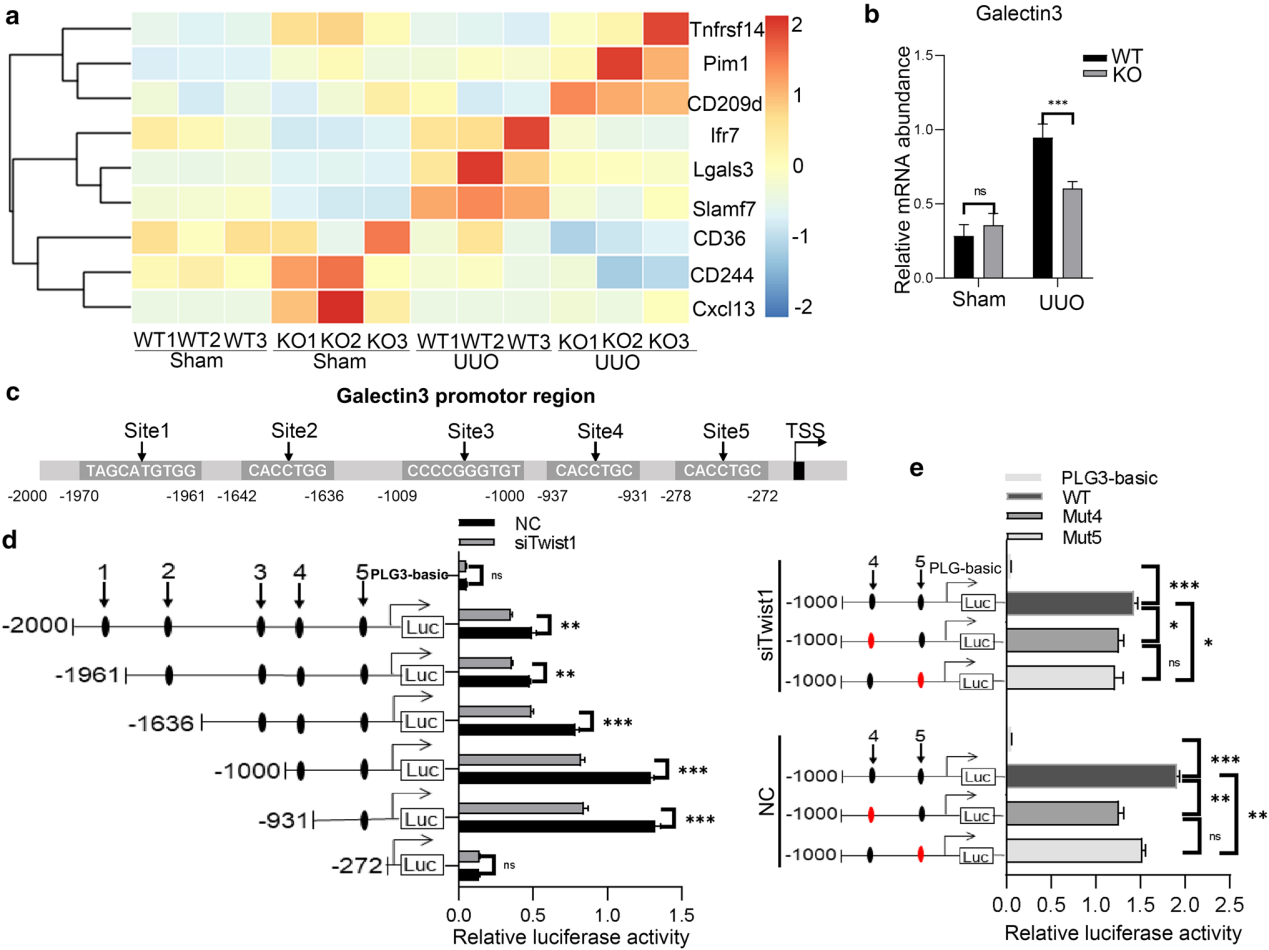
Galectin-3 is a direct target of Twist1. **a** mRNA gene sequence profiling of F4/80^+^ renal macrophages pooled from five mice, *n* = 3 independent experiments. **b** Real-time PCR analysis showing the mRNA abundance for galectin-3 in macrophages from sham and UUO kidneys at 14 days after surgery. Each sample was pooled from five animals within the same group. Macrophages from *Cre* + *Twist1fl/fl* versus macrophages from wild-type littermate UUO kidneys, *n* = 3 independent experiments. **c** Diagram of the galectin-3 promoter-luciferase reporter constructs containing the wild-type binding sites. **d** Serially truncated galectin-3 luciferase reporter constructs were transfected into Raw264.7 siRNA-twist1 (siTwist1) and Raw264.7 siRNA-Control (Control) cells. Luciferase activity values were measured and analyzed. Luciferase values were normalized to the empty vector control. siRNA-Twist1 versus empty vector controls, *n* = 3 independent experiments. **e** Relative luciferase activity in Raw264.7 cells co-transfected with wild-type or mutated reporter plasmids of galectin-3 or controls. Luciferase values were normalized to the empty vector control. siRNA-Twist1 versus empty vector controls, *n* = 3 independent experiments. *P < 0.05, **P < 0.01. Data are presented as the mean ± SEM. Data were first analyzed for normal distribution, and if data passed normality test, two-tailed Student’s *t* test for two groups and two-way ANOVA for multiple groups was used

**Fig. 9:**
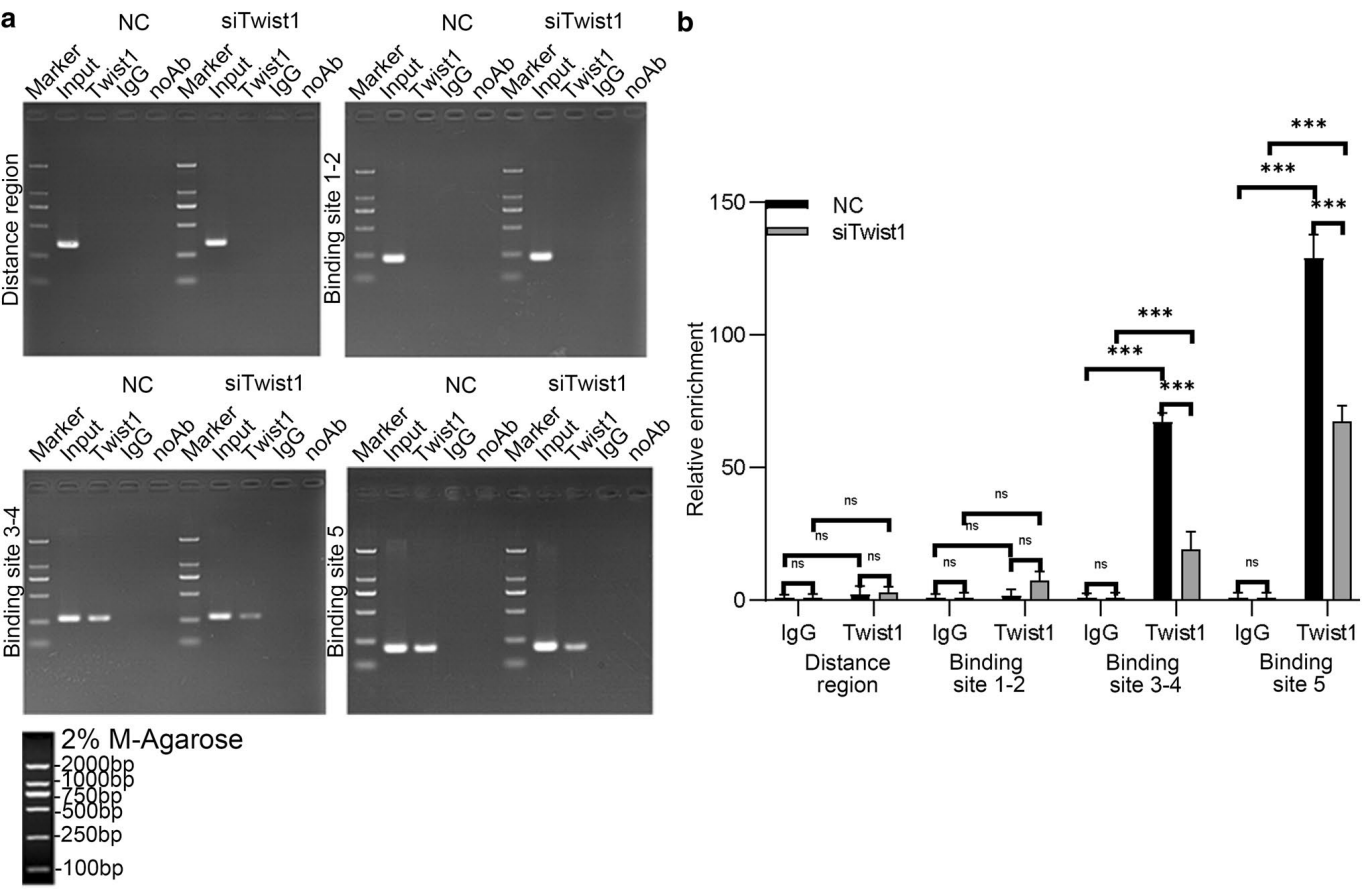
Twist1 transcribe Galectin-3 directly. **a** The chromatin immunoprecipitation assays demonstrated the direct binding of Twist1 to binding site 3–5 of the galectin-3 promoter in Raw264.7 cells. **b** Bar graphs are presented as the relative enrichment normalized to control IgG, *n* = 3 independent experiments. *P < 0.05, **P < 0.01. Data are presented as the mean ± SEM. Data were first analyzed for normal distribution, and if data passed normality test, two-tailed Student’s *t* test for two groups and two-way ANOVA for multiple groups was used

To further investigate the regulatory cellular pathways of Twist1 of inflammatory macrophage in kidney injury, we performed RNA sequencing (RNA-Seq) for transcriptional profiling of infiltrating F4/80^+^ macrophages from WT and *Lyz2-Cre* + *Twist1fl/fl* mice before and after UUO and prioritized the nine most differentially expressed genes (Fig. 8a and PRJNA648848). Of these, galectin-3 (lgals3) was highly expressed in F4/80^+^ macrophages from fibrotic kidneys of wild-type littermates, and was significantly reduced in macrophages from *Lyz2-Cre* + *Twist1fl/fl* mice on day 14 after UUO. The expression pattern of galectin-3 was further confirmed by RT-PCR in enriched macrophages from fibrotic kidneys (Fig. 8b). To further clarify the transcriptional activation of Twist1 on galectin-3, five putative Twist1-binding sites were predicted in the galectin-3 promoter region that could affect transcriptional activation (Fig. 8c and Supplementary Information Fig. 5a). Compared with control cells, macrophages with Twist1 knockdown displayed reduced promoter activity for all the truncated fragments, and site-directed serial deletion analysis of the galectin-3 promoter identified that Twsit1-binding site 4–5 (− 937 bp to − 272) was critical for Twist1-mediated transcriptional activation (Fig. 8d and Supplementary Information Fig. 5b). To confirm the significance of the binding sites 4–5 in regulating galectin-3, we mutated the binding sites 4 and 5 of the galectin-3 promoter (Supplementary Information Fig. 5c). As expected, mutating the binding sites 4 and 5 showed significantly reduced promoter activity compared with WT, but no clear changes were observed in Raw264.7 cells with Twist1 silencing (Fig. 8e). Furthermore, we identified a strong galectin-3 DNA band of 737 bp containing the Twist1 binding sites 3–5 in the promoter region (− 1009 to − 272) of galectin-3 in Twist1-enriched immunoprecipitates in Raw264.7 cells (Fig. 9a, b). A similar but weaker band was observed for these binding sites in Raw264.7 cells with Twist1 knockdown. No bands were evident in the other two possible binding sites (1 and 2) and the control IgG immunoprecipitates. These data provide evidence that binding sites 4 and 5 (− 1000 to − 272) in the promoter region of galectin-3 are critical for Twist1-induced galectin-3 activation.

### Decreased expression of galectin-3 in UUO renal tissues and macrophages of conditional Twist1-deficient mice

**Fig. 10:**
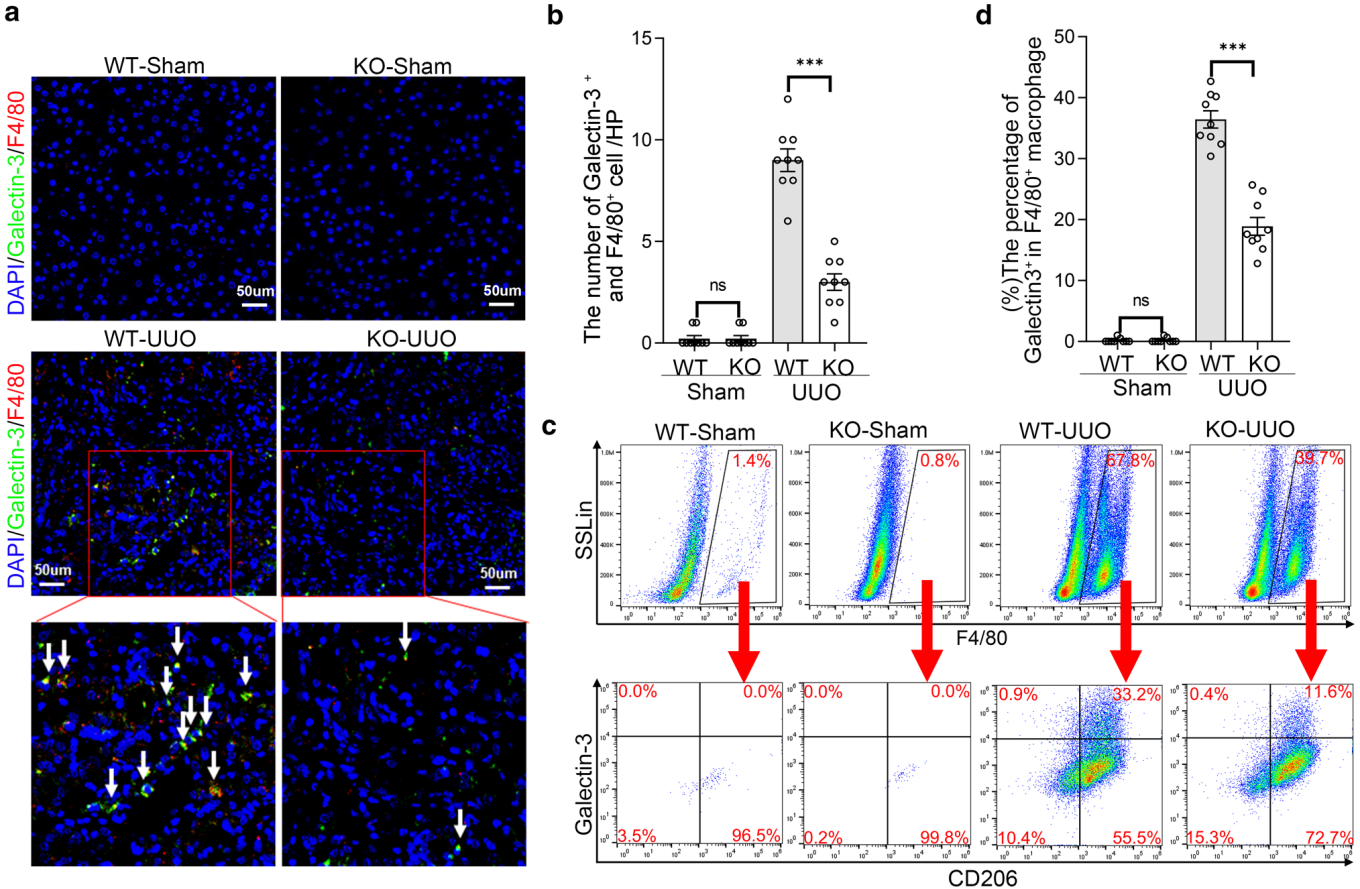
Decreased expression of galectin-3 in UUO renal tissues and macrophages of conditional Twist1-ablated mice. **a** Representative Immunostaining (× 40) of Galectin-3 (green) and macrophage (red; F4/80) expression in 3 µm kidney sections and **b** bar graph analysis demonstrating galectin-3 expression in macrophage as indicated kidney in four groups of mice. Bar scale = 50 µm. *n* = 3 animals per group, *n* = 3 micrographs analyzed per mouse, WT-UUO vs. KO-UUO. **c** Flow cytometry analysis of galectin-3 expression in F4/80^+^ CD206^+^ from renal tissue at 14 days after UUO. **d** Bar graph analysis of fibrotic kidney macrophage infiltration in renal tissue *Cre* + *Twist1fl/fl* at 14 days after UUO versus macrophages from wild-type littermate UUO kidneys, *n* = 3 animals per group. *P < 0.05, **P < 0.01. Data are presented as the mean ± SEM. Data were first analyzed for normal distribution, and if data passed normality test, two-tailed Student’s *t* test for two groups and two-way ANOVA for multiple groups was used

**Fig. 11:**
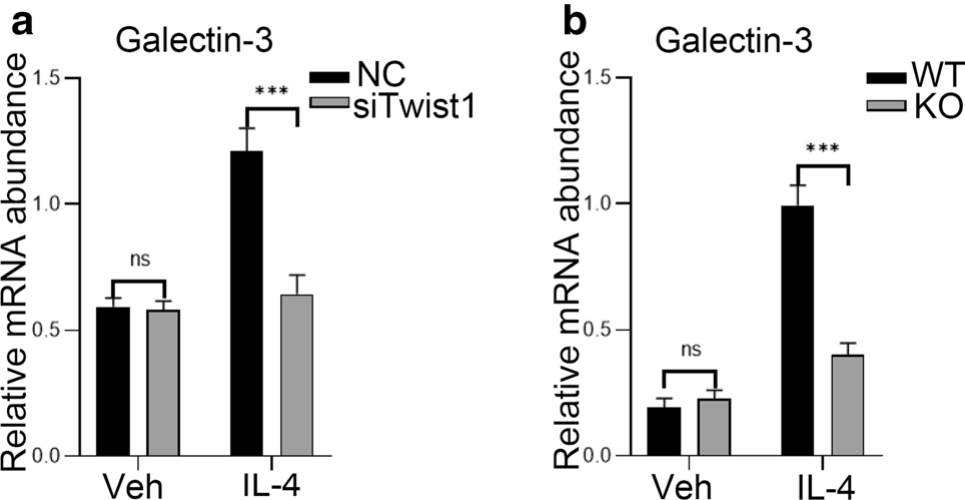
Knockdown Twist1 reduced expression of galectin-3 in macrophage. **a** Real-time PCR analysis of the mRNA abundance for galectin-3 in Twist1-silenced Raw264.7 cells exposed to IL-4, siRNA-Twist1 versus empty vector controls, *n* = 3 independent experiments. **b** Real-time PCR analysis of the mRNA abundance for galectin-3 in BMMs from *Cre* + *Twist1fl/fl* mice and wild-type littermate with galectin-3 upregulation. *P < 0.05, **P < 0.01. Data are presented as the mean ± SEM. Data were first analyzed for normal distribution, and if data passed normality test, two-tailed Student’s *t* test for two groups and two-way ANOVA for multiple groups was used

Galectin-3 plays an important disease-exacerbating role in autoimmune/inflammatory and cancer [36]. To explore the function of galectin-3 in macrophages transcribed by Twist1 during kidney fibrosis, we found that the expression of galectin-3 in the renal macrophages of *Lyz2-Cre* + *Twist1fl/fl* mice using co-immunofluorescence staining with F4/80 (Fig. 10a, b). Consistently, flow cytometry demonstrated that the galectin-3^high^CD206^+^ population was lower in renal macrophages of *Lyz2-Cre* + *Twist1fl/fl* mice on day 14 after UUO compared with controls (Fig. 10c, d). In vitro, RT-PCR showed that galectin-3 expression was downregulated in Twist1 knockdown Raw264.7 cells and isolated BMMs from myeloid-specific Twist1-deficient mice treated with IL-4 (Fig. 11a, b). Together, these results demonstrate that downregulation of Twist1 in macrophage has less galectin-3 expression during kidney fibrosis progression.

### Galectin-3 modulates Twist1-mediated M2 macrophage polarization

**Fig. 12:**
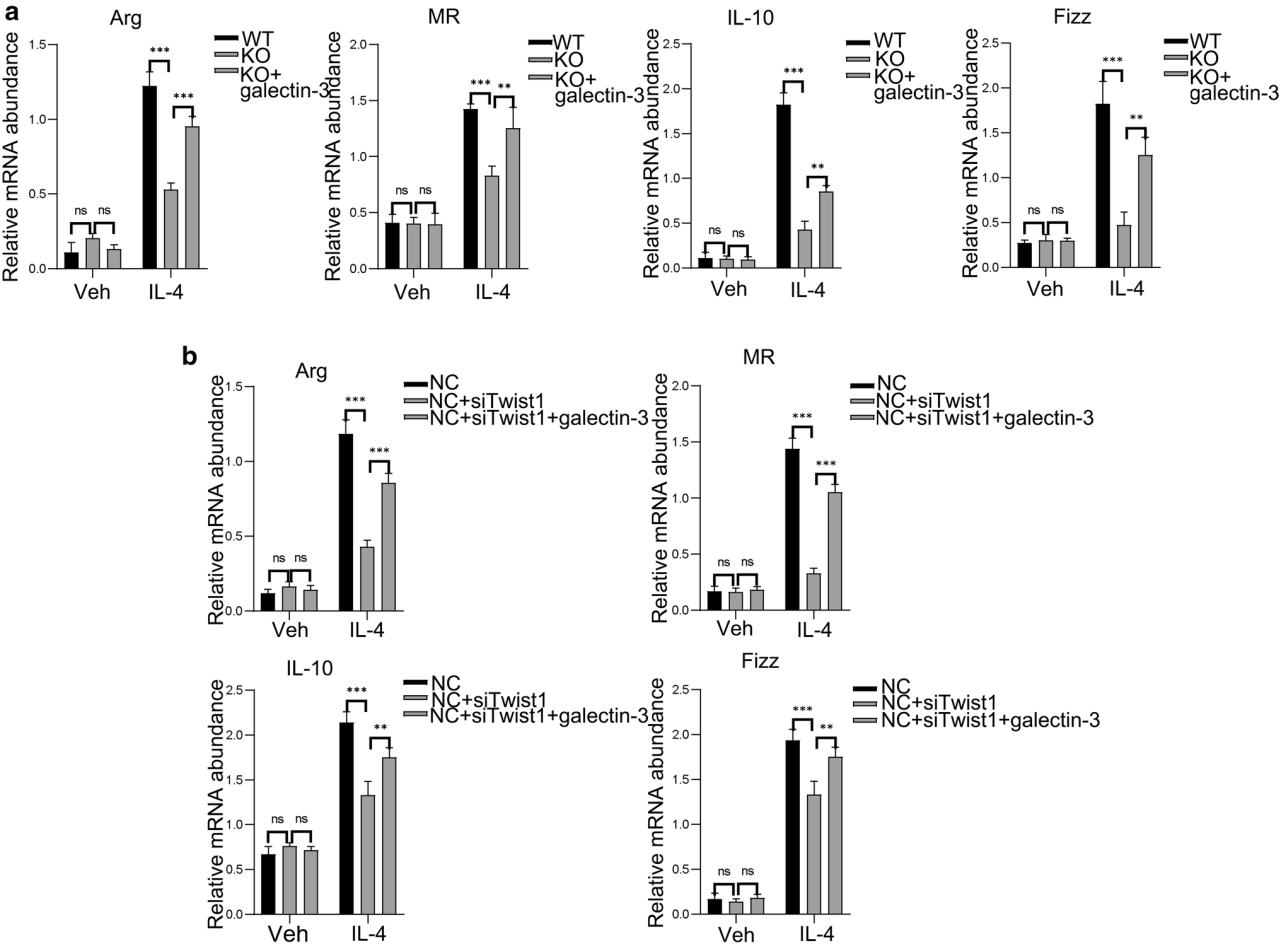
Galectin-3 modulates Twist1-mediated M2 macrophage polarization. **a** Relative mRNA abundance for Arg-1, MR, IL-10, and Fizz1 in BMMs from *Cre* + *Twist1fl/fl* mice with galectin-3 upregulation, BMMs from *Cre* + *Twist1fl/fl* mice with galectin-3 upregulation versus *Cre* + *Twist1fl/fl* mice without galectin-3 upregulation, *n* = 3 animals per group, *n* = 3 independent experiments. **b** Relative mRNA abundance for Arg-1, MR, IL-10, and Fizz1 in Twist1-silenced and galectin-3-upregulated Raw264.7 cells, siRNA-Twist1 with galectin-3 upregulation versus siRNA-Twist1 without galectin-3 upregulation, *n* = 3 independent experiments. Data in **a** and **b** were compared by two-tailed Student’s *t* test. *P < 0.05; **P < 0.01; ***P < 0.001. Data are presented as the mean ± SEM

To further identify the role of galectin-3 in macrophage polarization regulated by Twist1, we constructed a galectin-3 plasmid vector and was transfected in Twist1 silencing Raw264.7 and BMMs from *Lyz2-Cre* + *Twist1fl/fl* mice, we found galectin-3 overexpression partially rescued the reduced expression of the M2-associated genes Arg-1, MR, IL-10, and Fizz1 in macrophages (Fig. 12a). The reduced expression of the M2-associated genes Arg-1, MR, IL-10, and Fizz1 in Twist1 in Raw264.7 cells was consistently recovered by galectin-3 overexpression (Fig. 12b). Together, these data highlight a key role of the galectin-3 in M2 polarization.

## Discussion

We previously demonstrated that Twist1 expression in renal tubular epithelial cells plays an important role in EMT in renal fibrosis [37, 38]. In this study, we demonstrated for the first time that Twist1 is also highly expressed in renal macrophages of human kidneys with fibrotic renal disease and in the UUO mouse model. The study provides evidence that Twist1 in macrophages may regulate macrophage plasticity and heterogeneity to promote renal fibrosis. The ablation of Twist1 in macrophages significantly alleviated renal fibrosis in the UUO mouse model by inhibiting macrophage chemotaxis and M2 polarization, confirming that Twsit1-mediated macrophage heterogeneity plays a key role in renal fibrosis. Mechanistically, we found that Twist1 in macrophages might contribute to renal fibrosis through either the secretion of profibrotic growth factors or direct transition to myofibroblast-like cells. We identified galectin-3 as a direct target of Twist1 that can modulate Twist1-mediated M2 macrophage polarization.

Twist1, as a member of the basic helix–loop–helix family of transcription factors, has multiple functions that are associated with the pathogenesis of fibrotic diseases and tumor progression [39]. Twist1 signaling is relatively silenced in adult kidneys but can be reactivated in various experimental animal models and in CKD in humans [40]. Indeed, we found that Twist1 activation was increased in kidneys from IgAN patients with Lee's grade III–V, but was rarely expressed in IgAN patients with Lee's grade I–II (early stage of IgAN, no fibrosis). The role of activated Twist1 in renal disease remains unclear, although recent studies have linked Twist1 with renal fibrogenesis [41]. Twist1 is highly expressed in the tubular epithelia of the expanded tubules and interstitial areas of UUO kidneys and has been shown to be involved in tubular EMT, myofibroblast proliferation, and subsequent fibrosis in obstructed kidneys [42]. Furthermore, we reported that Twist1 is highly expressed in HK2 cells and promotes renal fibrosis by regulating EMT [37]. Recent evidence suggests that Twist1 signaling participates in kidney injury/repair [43]. Nevertheless, it was still unknown whether Twist1 regulates the biological functions of matrix cells that influence renal fibrosis.

Recent studies suggested that tissue-infiltrating and resident macrophages that accumulate at the site of renal injury contribute to renal fibrosis through differentiating into either M1 or M2 phenotypes on stimulation [44, 45], but the molecular mechanisms underlying the macrophage polarization in renal fibrosis remain unknown. Here, we provide evidence that increased Twist1 might be one of the mechanisms for the infiltration and functional heterogeneity of macrophages in renal fibrosis. Indeed, Twist1 expression in macrophages was positively correlated with severe renal fibrosis in patients with IgAN and renal fibrosis in mice after UUO. Nevertheless, the increased macrophage infiltration was abolished in the kidneys of mice with myeloid-specific Twist1 gene ablation. In addition, reduced levels of CCL2 released from and reduced expression of CCR2 in Twist1-deficient macrophages suggests the possibility that the CCL2/CCR2 chemotaxis axis might be one of the major pathways that contribute to the Twist1-mediated macrophage infiltration in UUO kidneys. In addition, CXCL1/CXCR1, CXCL16, CCL5, macrophage migration inhibitory factor (MIF) and Osteopontin are associated with the recruitment of macrophages from bone marrow. Thus, it remains possible that those molecules might also be involved in Twist1-mediated macrophage infiltration.

We also made a novel finding that Twist1 in macrophages modulates macrophage polarization. M1 macrophages produce large amounts of proinflammatory mediators, while M2 macrophages exhibit anti-inflammatory features and are involved in renal repair and fibrosis [46, 47]. We found that there was a time-dependent increase in the percentage of M2 macrophages that may contribute to renal fibrosis after UUO. Of interest, the increased M2 macrophages were reduced in mice with Twist1-ablated macrophages (*Lyz2-Cre* + *Twist1fl/fl*), implying that Twist1 is critical in macrophage polarization towards M2 or maintaining M2 phenotypes. This was indeed supported by our in vitro analysis that Twist1 silencing in macrophages clearly reduced the expression of M2-associated genes, whereas no changes were noted for M1-associated genes.

We explored the mechanism of the contribution of Twist1-regulated M2 macrophages in renal fibrosis. M2 macrophages promote renal fibrosis through the secretion of profibrotic growth factors or direct transition to myofibroblast-like cells. Thus, we analyzed the expression of profibrotic cytokines and found that most of these highly selected profibrotic cytokines, such as PDGF, VEGF, and TGFβ, were increased in enriched M2 macrophages from fibrotic kidneys. Intriguingly, these cytokines were lower in Twist1-deficient M2 macrophages, suggesting that Twist1 modulates the expression of profibrotic cytokines that might lead to the development of renal fibrosis. We explored the possibility that Twist1 might modulate the macrophage transition to myofibroblast-like cells. We found that levels of fibrotic protein, including Col-1 and α-SMA, were lower in Twist1-ablated BMMs from fibrotic kidneys of *Lyz2-Cre* + *Twist1fl/fl* mice and in Twist-silenced Raw264.7 cells, indicating that Twist1 modulates macrophage transition to myofibroblast-like cells. Those results suggest that Twist1 plays an important role in M2 macrophages involved in renal fibrosis through controlling the secretion of profibrotic growth factors or direct transition to myofibroblast-like cells.

A previous study showed that HIF-1α, Snail, and other upstream signal molecules induced by injury regulate Twist1 transcriptional activation in renal disease [37, 48]. We previously demonstrated that Twist1 is involved in hypoxia-induced EMT and contributes to fibrogenesis in renal tubular cells by HIF-1α activation [37], but it was still unclear whether downstream molecules regulated by Twist1 could promote renal fibrosis. Using RNA-seq, we identified galectin-3 as a direct target of Twist1. The expression of galectin-3 was correlated with Twsit1 in macrophages of mouse models after UUO or enriched macrophages from fibrotic kidneys. We then identified five putative Twist1 binding sites in the galectin-3 promoter region. The luciferase reporter assay demonstrated that two binding sites showed increased promoter activity. This finding was further supported by chromatin immunoprecipitation assays. A strong DNA band containing these two binding sites was identified in Twist1-enriched immunoprecipitates by Twist antibody in macrophages. These data suggest that galectin-3 might be a direct downstream target that contributes to Twist1-mediated macrophage polarization and renal fibrosis.

Mounting evidence indicates that galectin-3 is highly expressed and secreted by macrophages and drives alternative macrophage activation in myocardial repair after myocardial infarction [49], activates a variety of profibrotic factors, promotes fibroblast proliferation and transformation, mediates collagen production, and exhibits profibrogenic functions in chronic diseases [50]. Elevated galectin-3 levels have been reported in fibrotic conditions affecting the heart [51], liver [52], blood vessels [53], and lungs [54], In our study, we found that galectin-3 was regulated by Twist1, and provided additional evidence that galectin-3 is also involved in kidney fibrosis.

In conclusion, Twist1 signaling exacerbates IL-4-induced macrophage M2 polarization through galectin-3 induction. Future studies will determine whether galectin-3 is associated with CKD by examining galectin-3 expression in fibrotic kidneys and its role in macrophage polarization and development of renal fibrosis. It is likely that other Twist1 downstream targets might also be involved in Twist1-mediated macrophage polarization and renal fibrosis. Persistent chronic injury leads to the accumulation of M2 macrophages in a progressive kidney fibrosis model induced by UUO. Importantly, Twist1 plays an important role in macrophage infiltration and macrophage polarization in the kidney of UUO models. Galectin-3 is a downstream target of Twist1 that contributes to the Twist1-mediated macrophage polarization. Thus, targeting Twist1 and its target galectin-3 might be a new strategy for delaying kidney fibrosis in patients with CKD. Metformin, a Twist inhibitor [55], reduced M2 macrophages infiltration in UUO kidneys [56, 57]. However, Metformin will affect the immune system including monocytes, B cells, and T cells in the kidney as well as in the peripheral blood and spleen after UUO, the protection effect of immunity cells will be weakened because of limitation of function [58]. Based on our research, Twist inhibitors (Metformin) targeting macrophage specifically will be possible therapeutics that could be used for CKD.

## Materials and methods

### Human kidney biopsies

gA nephropathy (IgAN) is the most common type of chronic kidney disease (CKD) and one of the major causes of renal fibrosis [30]. Hence, we chose the IgA nephropathy patients samples to investigate the relationship between Twist1 and renal fibrosis. Renal biopsy samples and Clinical data from patients diagnosed with IgAN at the Xijing Hospital (Xi’an, China) are shown in Supplementary Information Table 2. Histological examination was performed at the Kidney Pathology Department of Xijing Hospital. The relevant clinical information was collected from patients’ records.

### Animal model

Male C57BL/6 mice weighing 20 ± 2 g were acquired from the specific pathogen-free laboratory animal center of the Fourth Military Medical University utilizing a 14-h light and 10-h dark cycle and maintained according to the guidelines of the Institutional Animal Care and Use Committee at Fourth Military Medical University. UUO was performed as previously reported [59]. The mice were euthanized, kidneys and bone marrow were harvested at days at 0, 3, 7, and 14 after UUO.

Homozygous Twist1 floxed mice [*B6; 129S7-Twist1tm2Bhr/Mmnc*] and mice expressing the Cre fusion protein under the control of macrophage-specific mouse Lyz2 promoter were acquired from Jackson Laboratories (West Grove, PA, USA). All animals were housed in the specific pathogen-free laboratory animal center of Fourth Military Medical University, as described above. Mating Twist1 floxed mice with *Lyz2-Cre* transgenic mice generated mice that were heterozygous for the Twist1 floxed allele (genotype: *Lyz2-Cre*+*, Twist1fl/wt*). These mice were crossbred with homozygous Twist1 floxed mice (genotype: *Lyz2-Cre*+*; Twist1fl/fl*) to generate offspring with different littermates (*Lyz2-Cre*+; *Twist1fl/fl, Lyz2-Cre*+; *Twist1fl/wt, Lyz2-Cre−*; *Twist1fl/wt*, and *Lyz2-Cre−*; *Twist1fl/fl)*. *Lyz2-Cre*+; *Twist1fl/fl* mice and the same-sex *Lyz2-Cre−*; *Twist1fl/fl* littermates (controls) were subjected to UUO. The sham group underwent the same procedure but without UUO. Genotyping was performed by PCR assay using DNA extracted from the mouse tail and using the following primers: Cre transgene, sense: 5′-CCGGTCGATGCAACGAGTGATGAGG-3′; antisense: 5′-GCCTCCAGCTTGCATGATCTCCGG-3′; Twist1 floxed, sense: 5′-AGCGGTCATAGAAAACAGCC-3′; antisense: 5′-CCGGATCTATTTGCATTTTACCATGGGTCATC-3′.

### Cell culture

Raw264.7 cells were cultured in RMPI-1640 containing 20% (vol/vol) FBS (GIBCO, Invitrogen Inc, Carlsbad, CA, USA) and 1% (vol/vol) antibiotics (100 U/ml penicillin) at 37 °C in 5% CO_2_. Raw264.7 stimulated with IL-4 (25 ng/ml; catalog no. 214-14; PeproTech, Rocky Hill, NJ, USA), or with IFN-γ (25 ng/ml; catalog no. 315-05; PeproTech, Rocky Hill, NJ, USA) and LPS (100 ng/ml; catalog no. L2630; Sigma, St Louis, MO, USA) for 24 h. Adherent cells were washed and harvested with trypsin/EDTA (Lonza).

Bone marrow-derived macrophages (BMMs) were isolated, as previously described [60]. BMMs obtained from *Lyz2-Cre* + *Twist1fl/fl* and *Lyz2-Cre-Twist1fl/fl* mice were cultured in RMPI-1640 containing 10% (vol/vol) FBS, 25 ng/ml mouse M-CSF (catalog no. 315-02; PeproTech, Rocky Hill, NJ, USA), and 1% (vol/vol) penicillin/streptomycin antibiotics for 5 days, Briefly, on day 5, cells were replated in triplicate (3 × 105 cells/well). BMMs were cultured with serum-free medium and treated with IL-4 (25 ng/ml; catalog no. 214-14; PeproTech, Rocky Hill, NJ, USA), or with IFN-γ (25 ng/ml; catalog no. 315-05; PeproTech, Rocky Hill, NJ, USA) and LPS (100 ng/ml; catalog no. L2630; Sigma, St Louis, MO, USA) for 24 h. Adherent cells were washed and harvested with trypsin/EDTA (Lonza).

### Semiquantitative analysis of the fibrotic area in kidney tissue

Mouse kidney sections of 3 µm in thickness were stained with the Masson Trichrome kit (catalog no. HT15-1KT; Sigma, St Louis, MI, USA), according to the manufacturer’s protocol. Accumulated collagen in the interstitial area was stained with aniline blue. Ten × 400 fields were randomly selected in the cortical area for each kidney section. The percentage of interstitial fibrotic area to the selected field was analyzed with Image-Pro Plus 6.0 (Media Cybernetics, Inc., Rockville, MD, USA), and an average percentage of fibrotic kidney area for each section was calculated.

### Histology and immunohistochemistry

Paraffin-embedded mouse kidney sections (3-µm thickness) were stained with PAS (catalog no. G1280; Solarbio, Beijing, China), Masson (HT15-1KT; Sigma–Aldrich, St Louis, MI, USA), and Sirius red (catalog no. G1472-2; Solarbio, Beijing, China). The antibodies for immunohistochemistry were: anti-α-SMA (catalog no ab32575, Abcam, Cambridge, United Kingdom), anti-fibronectin (catalog no. 610154; Transduction Laboratories, Lexington, KY, USA), anti-type I collagen (catalog no. AB765P; Millipore Corp., Billerica, MA, USA) and anti-CCR2 (catalog no. ab176390; Abcam, Cambridge, United Kingdom) and anti-galectin-3 (catalog no. 3027070; Millipore Corp., Billerica, MA, USA). After incubation with the primary antibodies at 4 °C overnight, the slides were stained with the secondary antibody for 1 h at room temperature. The sections were incubated with the ABC reagents for 1 h at room temperature before DAB staining (Vector Laboratories, Burlingame, CA, USA). Images were captured using a light microscope (Olympus, Tokyo, Japan).

### Immunofluorescence

Kidney cryosections at 3 µm thickness were fixed for 15 min with 4% paraformaldehyde followed by permeabilization with 0.3% Triton X-100 in 1 × PBS for 5 min at room temperature. After blocking with 2% donkey serum for 60 min, the slides were stained with the following antibodies: anti-Twist1 (catalog no. 50581; Abcam, Cambridge, United Kingdom), anti-CD68 (catalog no. 31630; Abcam, Cambridge, United Kingdom),anti-F4/80 (catalog no. 6640; Abcam, Cambridge, United Kingdom), anti-cleaved caspase3 (catalog no. 9664; Cell Signaling Technology, Inc., Danvers, MA, USA), and anti-galectin-3 (catalog no. 3027070; Millipore Corp., Billerica, MA, USA), followed by staining with Alexa 488- or Cy3-conjugated secondary antibodies. For the quantitative analysis of Twist1 expression in macrophages in kidney tissues, ten × 400 fields were randomly selected in the cortical area from each kidney section.

### Kidney monocyte/macrophage enrichment

Mice were sacrificed by i.p. injection of Beuthanasia-D (Merck). After perfusion with cold 1 × PBS, the mouse kidneys were removed, minced into fragments, and digested in HBSS containing 1 mg/ml collagenase (catalog no. c5138; Sigma, St Louis, MI, USA) for 1 h 37 °C with intermittent agitation. The fragments were filtered through a 70 µm mesh (DKW33-N25, Dakewei, Shanghai, China) to achieve a single-cell suspension. RBC Lysis Buffer (eBioscience) was used to lyse RBCs at room temperature and cell counts were performed on the cell suspensions from the kidney digests. In some experiments, the kidney tissues were inflated with 10% formalin, removed, and fixed in 10% formalin prior to paraffin embedding. Sections were stained with H&E, Masson, PAS, Siris red. Macrophages were enriched from the single-cell suspension with F4/80 Microbeads and BD magnetic frame (BD, Bergisch-Gladbach, Germany), according to the manufacturer’s instruction.

### Quantitative real-time PCR

Total RNA was extracted using the RNeasy Plus Mini Kit (Qiagen, Hilden, Germany) according to the manufacturer’s instructions and RNA concentrations were determined using a NanoDrop 1000 (Thermo Fisher Scientific). Then, cDNA was synthesized using a PrimeScript RT reagent kit (TaKaRa, Dalian, China). The SYBR Premix Ex Taq II (TaKaRa) was used to amplify the double-stranded cDNA of interest. RT-PCR primers for Twist1, galectin-3, PDGFA, PDGFB, PDGFC, PDGFD, TGFβ1, TGFβ2, TGFβ3, VEGFA, CCN2, YM1, Fizz, and ACTB (β-actin) were purchased from Ruibo Bio (Guangzhou, China). RT-PCR primers for iNOS, IL-6, TGF-β, CCL2, TNF-α IL-1β, IL-10, Arg-1, and MR were synthesized by TaKaRa (Dalian, China). The levels of ACTB were used as internal controls for mRNA. The 2-ΔΔCt method was used to determine the relative expression level of RNA between groups. The primer sequences are listed in Supplementary Information Table 3.

### Protein isolation and western blots

Protein lysates were collected in RIPA lysis buffer (Beyotime, Shanghai, China) containing a complete protease inhibitor cocktail (Roche, Manheim, Germany). Lysates were centrifuged for 5 min at 14,000*g* to clear lysates and the supernatant was collected. Total protein was quantified by BCA assay following manufacturers protocols and 5–10 μg of total protein was used for each sample. 10 × Reducing Agent and 4 × LDS Sample Buffer and heated at 70 °C for 10 min. Bolt Bis–Tris gradient gels (4–12%) were used for electrophoresis and proteins were transferred onto 0.2-μm PVDF at 20 V for 75 min using Bolt transfer buffer containing 10% methanol. Wash buffer was TBS containing 0.05% Tween 20, and 5% BSA was added for blocking and incubation steps in primary and secondary antibodies. The proteins were visualized using a Dura Super Signal Substrate (Pierce Chemical, Dallas, TX, USA). Bands were detected by chemiluminescence using Supersignal West Femto (Pierce) on an Omega Ultra Lum imaging system. The blots were scanned using a Molecular Imager ChemiDox XRS + Imaging System with Image Lab software (Bio-Rad, Hercules, CA, USA).

The following antibodies were used: anti-Twist1 [#49254 (1:2000), Abcam, Cambridge, United Kingdom], anti-IL-10 [ab33471 (1:200), Abcam, United Kingdom], anti-YM1 [#60130 (1:1000), Stemcell Technologies Inc, Vancouver, Canada], anti-α-SMA [ab32575 (1:500), Abcam, Cambridge, United Kingdom], anti-type I collagen [AB765P (1:500); Millipore Corp, Billerica, MA, USA)] and anti-ACTN [β-actin (1:1000) ZSGB-BIO, Shanghai, China).

### Luciferase reporter assay

The mRNA 3′-UTR luciferase reporter vectors were constructed as previously described [61]. For the 3′-UTR luciferase reporter assays, the indicated cells were co-transfected with Galectin-3 promoter plasmids and pGL3-basic (RiboBio Co., Guangzhou, China) and the indicated wild-type using Lipofectamine 2000 (Thermo Fisher Scientific, Waltham, MA, USA). For luciferase reporter assays of promoter activity, the indicated cells were co-transfected with the pGL3-galectin-3 promoter fragments, pRL-SV40 Renilla lucifera2se reporter, and siRNA-Twist1 or control. The DualLuciferase Assay (Promega, Madison, WI, USA) was used to detect Renilla and firefly luciferase activities. Renilla luciferase activity was normalized to the firefly activity and presented as the relative luciferase activity. All assays were performed in triplicate three times.

### Chromatin immunoprecipitation (ChIP)

ChIP assays were performed as previously described [62]. Briefly, the recovered supernatants were incubated with a rabbit anti-Twist1 antibody (#50887, Abcam, Cambridge, United Kingdom) or an isotype control IgG (BD Biosciences, Franklin Lake, NJ, USA) for 2 h in the presence of herring sperm DNA and protein A/G magnetic beads. The DNA was recovered and subjected to PCR to amplify the Twist1-binding sites. The primers are shown in Supplementary Information Table 4.

### Plasmid construction

The galectin-3 promoter construct was generated as previously described [63]. Briefly, − 2000 to − 1 galectin-3 was generated from mouse genomic DNA. This construct, corresponding to the sequence from − 2000 to − 1 (relative to the transcriptional start site) of the 5′-flanking region of the mouse gene, was generated with the forward and reverse primers incorporating MluI and XhoI sites at the 5′ and 3′ ends, respectively. The MluI and XhoI sites of the pGL3-Basic Vector (Promega, Madison, WI, USA) were inserted for the ultimate PCR product. Constructs including a deletion of the 5′-flanking region of the galectin-3 promoter: (− 2000/− 1) galectin-3-1, (− 1961/− 1) galectin-3-2, (− 1636/-1) galectin-3-3, (− 1000/− 1) galectin-3-4, (− 931/− 1) galectin-3-5, and (− 272/− 1) galectin-3-6 and were generated in manner analogous to that for the (− 2000/− 1) galectin-3 construct. The QuikChange II Site-Directed Mutagenesis Kit (Stratagene, La Jolla, CA, USA) was used to generate the constructs for site-directed mutation. All constructs were verified by sequencing. All primers are listed in Supplementary Information Table 5.

### Oligonucleotide transfection

The sense strand sequences of the Twist1 siRNAs designed to target mouse cells were: Twist1 siRNA no. 738 (siRNA-1), 5′-CGGACAAGCUGAGCAAGAUTT-3′; Twist1 siRNA no. 780 (siRNA-2), 5′-GGUACAUCGACUUCCUGUATT-3′, and Twist1 siRNA no.832 (siRNA-3), 5′-GAUGGCAAGCUGCAGCUAUTT-3′. Successful knockdown of Twist1 was confirmed by western blotting (Supplementary Information Fig. 3a). Transfection of the siRNA was performed using the RNA iMAX Reagent (Thermo Fisher Scientific, Waltham, MA, USA), according to the manufacturer’s instructions.

### Flow cytometry

FACS analysis was performed according to previous reports [64]. Briefly, staining of cells for flow cytometry was performed in suspension cell from kidney tissue using between 1 × 10^5^ and 1 × 10^6^ cells per tube. For phospho-flow staining, kidney cells were resuspended in ice-cold PBS and immediately an equal volume of prewarmed BioLegend fixation buffer (catalog 420801, San Diego, CA, USA) was added and samples were incubated at 37 °C for 15 min. The BioLegend intracellular staining with True-Phos Perm Buffer (catalog 425401, San Diego, CA, USA) protocol was followed and all washes were performed with BioLegend Cell Staining Buffer (catalog 425401, San Diego, CA, USA). BioLegend Trustain FcX for mouse (clone 93, catalog 101320, San Diego, CA, USA) were used to block samples before staining. Phospho-flow experiments were collected on a BD LSRII and kidney homogenate analyses were collected using a BD FACS Canto RUO. FlowJo software (BD) was used for analysis of flow cytometry data.

The following antibodies were used: anti-CD86-PE (105007; Biolegend, San Diego, CA, USA), anti-F4/80-FITC (101205; Biolegend, San Diego, CA, USA), anti-CD206-PE (141720; Biolegend, San Diego, CA, USA), and anti-galectin-3-PECy7 (125418; Biolegend, San Diego, CA, USA).

### Statistical analyses

GraphPad Prism 8 software was used to perform statistical analyses and specific statistical tests used are listed in individual figure legends. Multiple *t* tests were performed with corrections for multiple comparisons using the Holm–Sidak method, while two-tailed unpaired *t* tests were used where indicated in figure legends. *P* < 0.05 was considered statistically significant and specific *P* value identifiers are listed in each figure legend. Some data sets were checked for statistical outliers using the GraphPad Prism outlier calculator with an α of 0.05; if a data point was determined to be a significant outlier it was not included in the graphs or when calculating statistical significance.

### Supplementary Information

Below is the link to the electronic supplementary material.

Supplementary file1 (TIF 52599 KB)

Supplementary file2 (TIF 31129 KB)

Supplementary file3 (TIF 30622 KB)

Supplementary file4 (TIF 29649 KB)

Supplementary file5 (TIF 29006 KB)

Supplementary file6 (DOCX 56 KB)

## Data Availability

All the data used for this study are presented in the paper or the Supplementary Materials.
