# Supplementary figures and images for: Twist1 regulates macrophage plasticity to promote renal fibrosis through galectin-3
Source: Cell Mol Life Sci. 2022 Feb 19;79(3):137. doi: 10.1007/s00018-022-04137-0 (PMC8858306; doi:10.1007/s00018-022-04137-0)

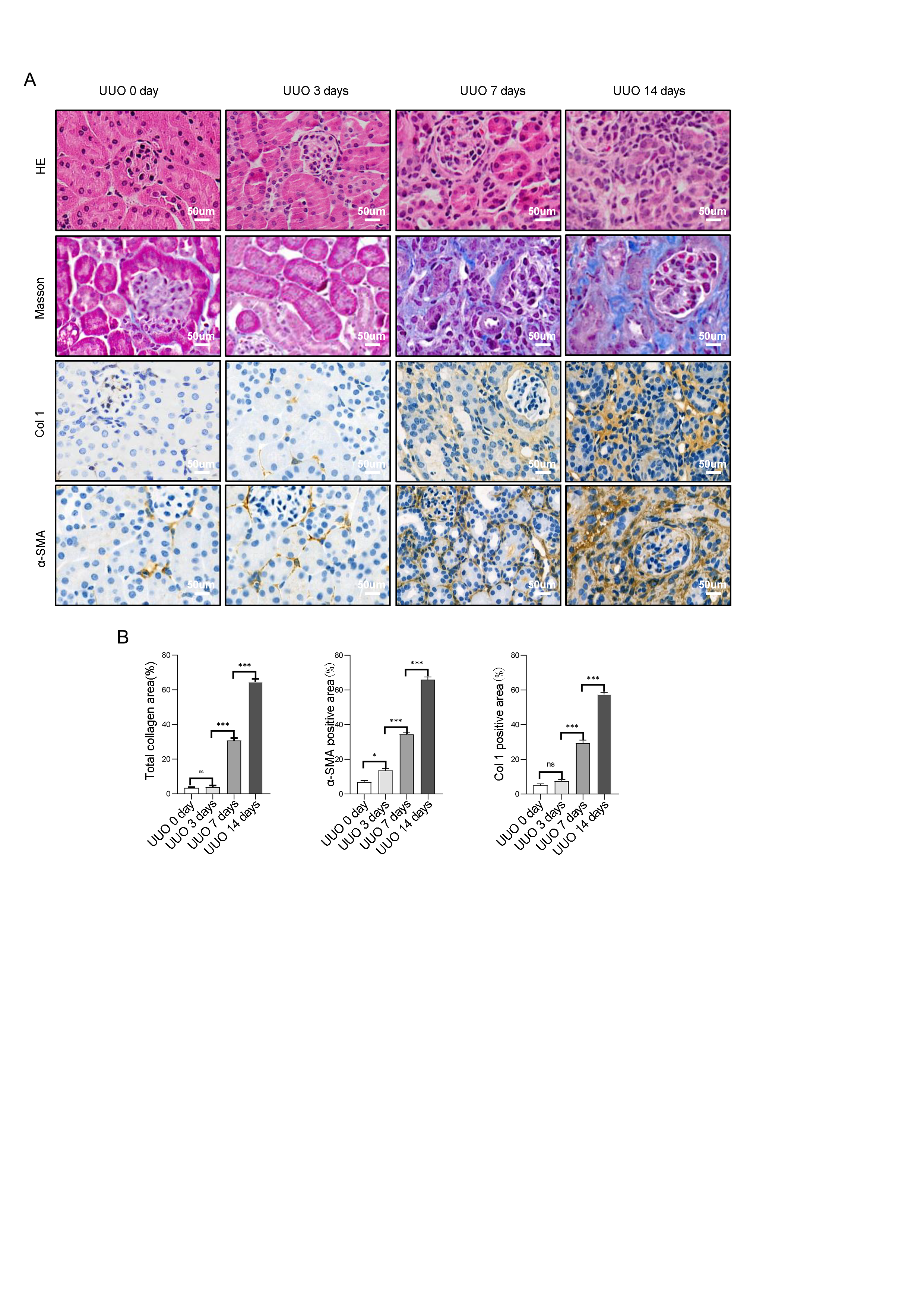

Supplement: Supplementary file 1 — Supplementary file1 (TIF 52599 KB) [file 18_2022_4137_MOESM1_ESM.tif]

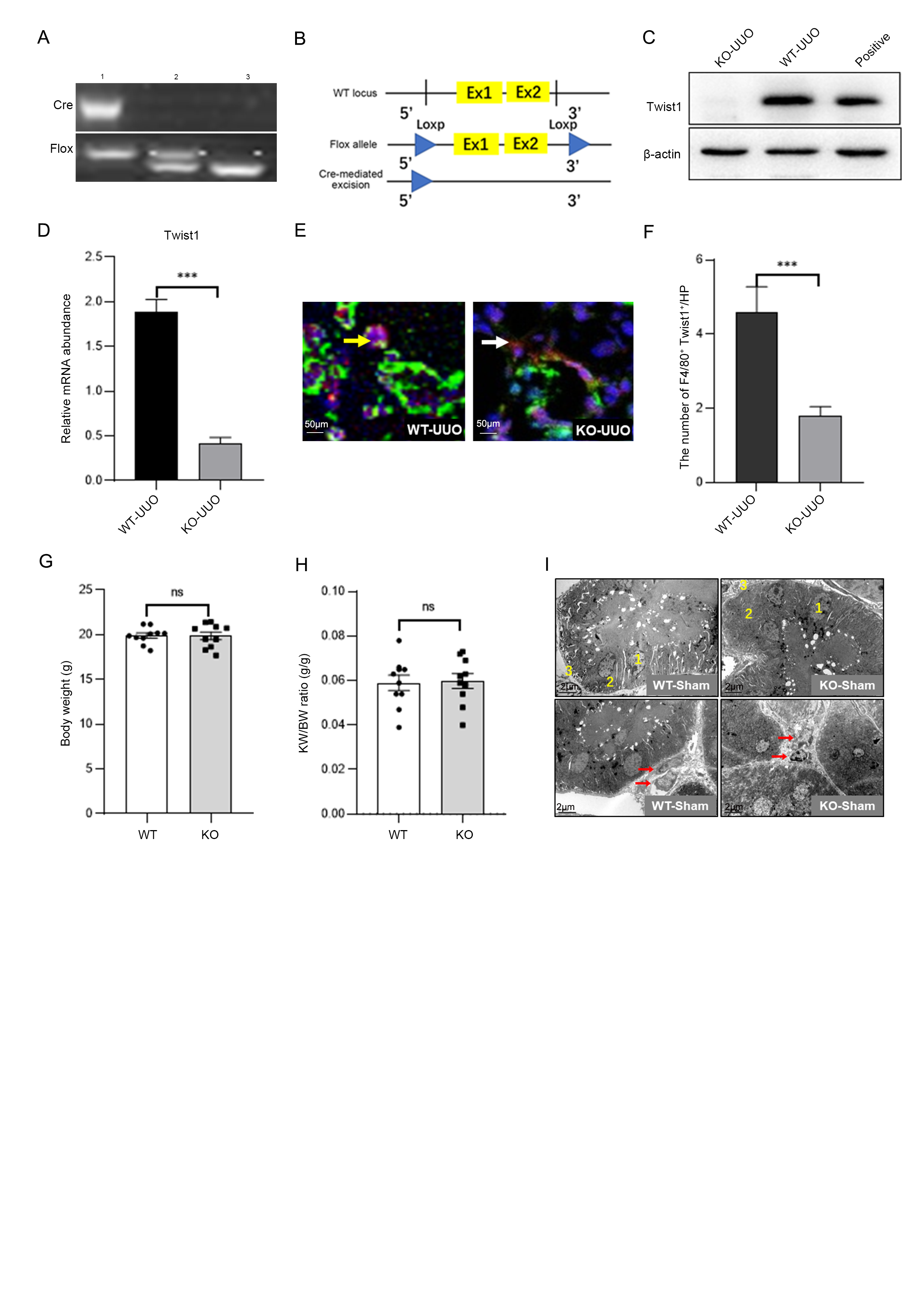

Supplement: Supplementary file 2 — Supplementary file2 (TIF 31129 KB) [file 18_2022_4137_MOESM2_ESM.tif]

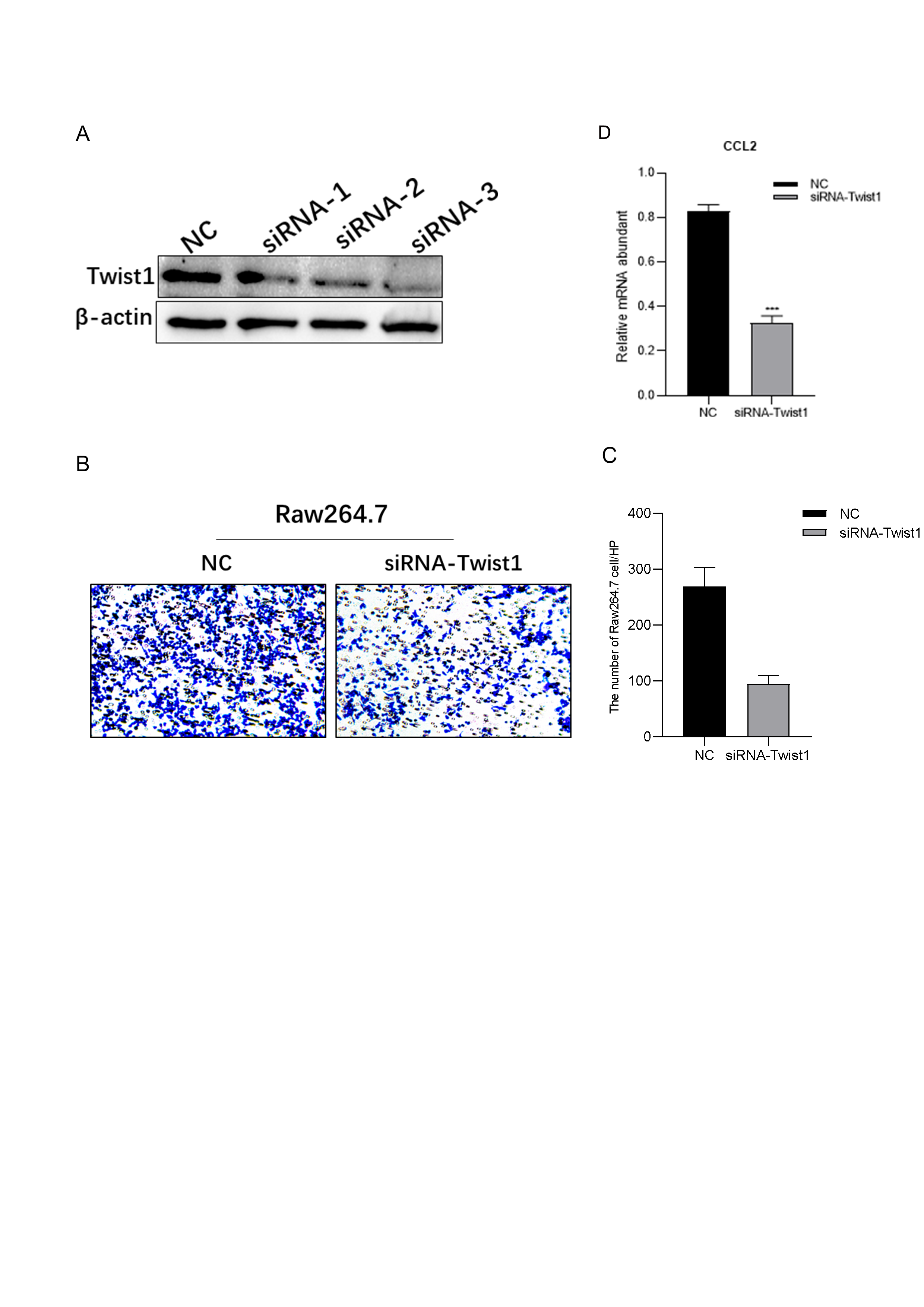

Supplement: Supplementary file 3 — Supplementary file3 (TIF 30622 KB) [file 18_2022_4137_MOESM3_ESM.tif]

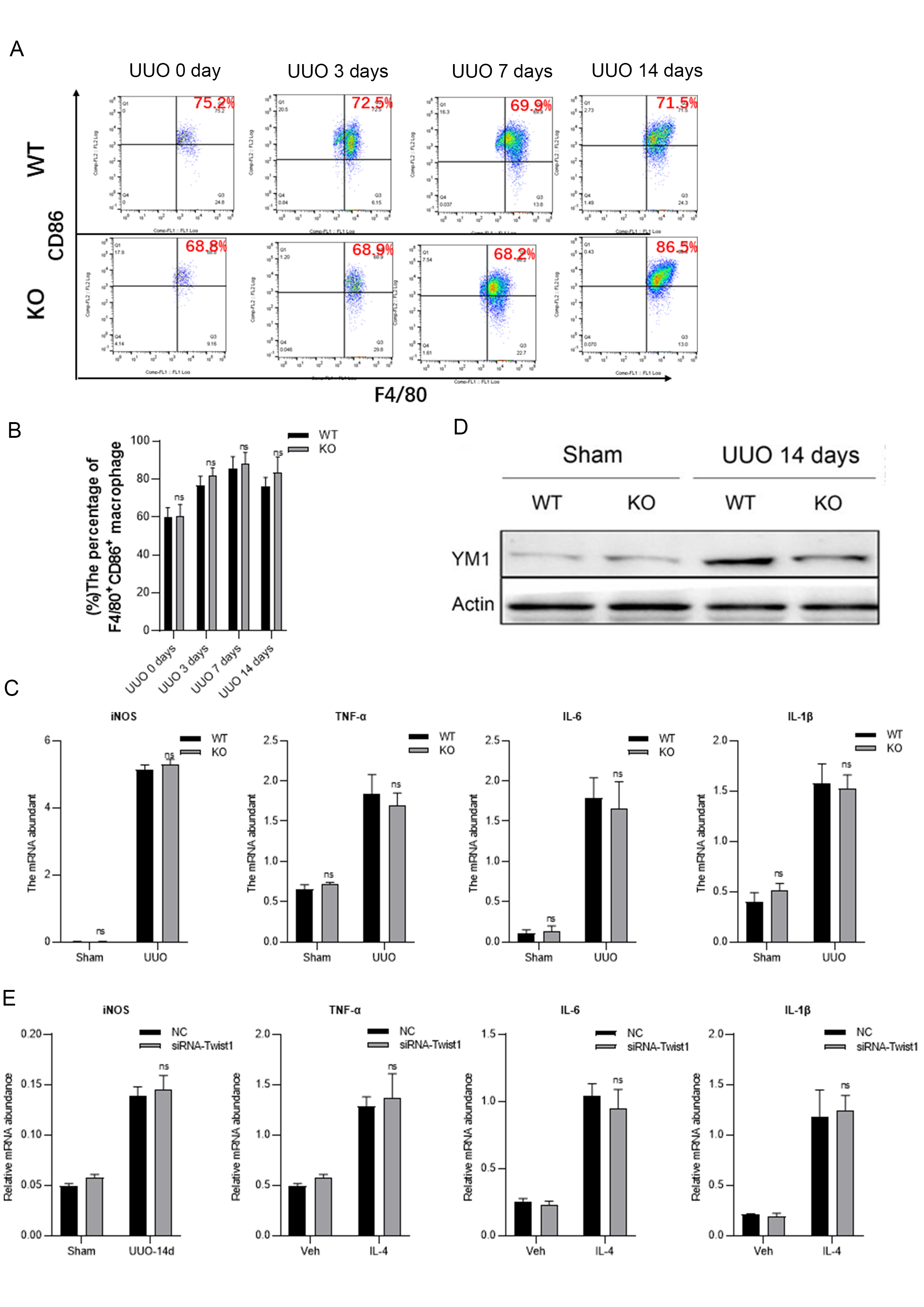

Supplement: Supplementary file 4 — Supplementary file4 (TIF 29649 KB) [file 18_2022_4137_MOESM4_ESM.tif]

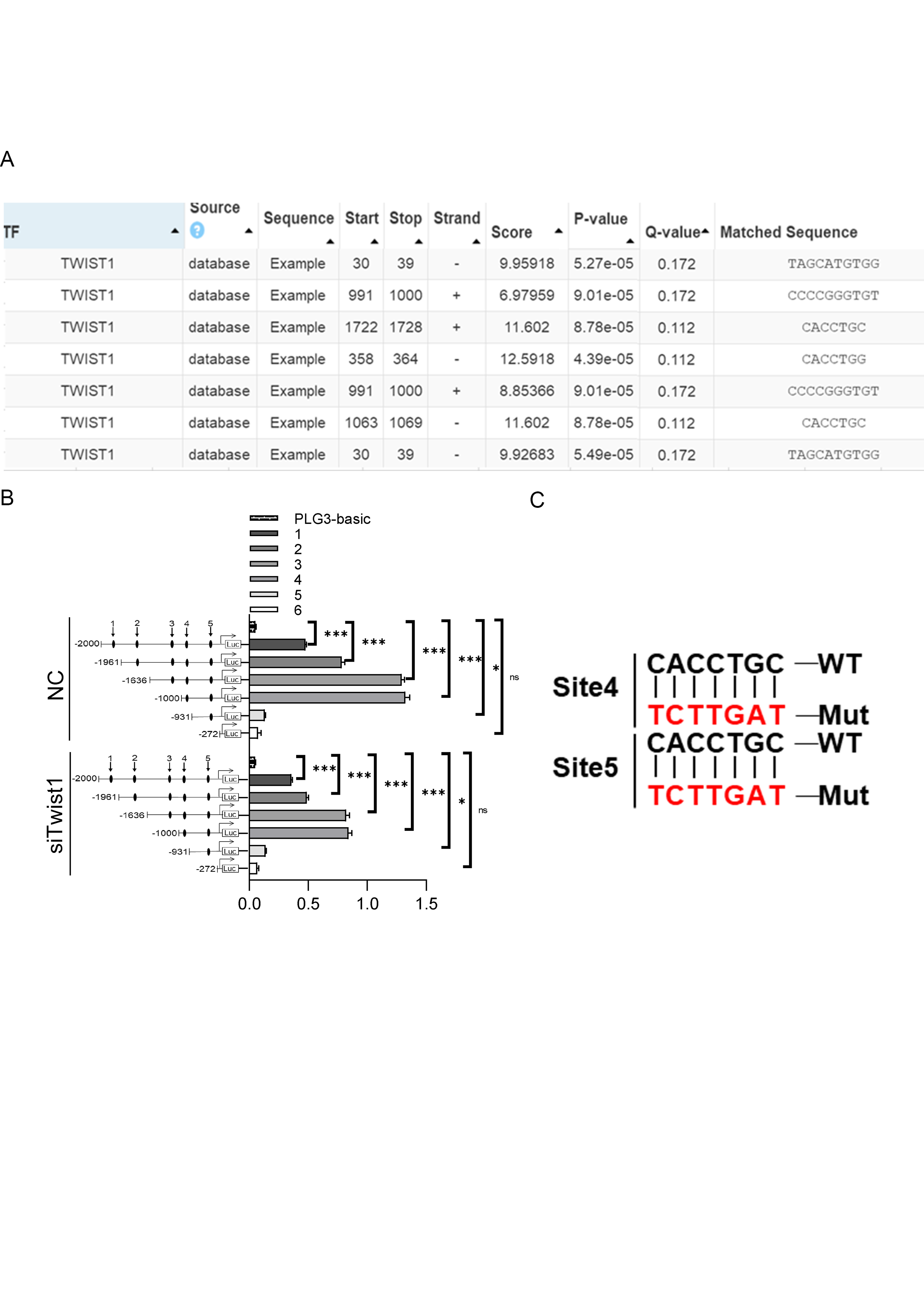

Supplement: Supplementary file 5 — Supplementary file5 (TIF 29006 KB) [file 18_2022_4137_MOESM5_ESM.tif]
