# Supplementary material for: Twist1 regulates macrophage plasticity to promote renal fibrosis through galectin-3
Source: Cell Mol Life Sci. 2022 Feb 19;79(3):137. doi: 10.1007/s00018-022-04137-0 (PMC8858306; doi:10.1007/s00018-022-04137-0)
Supplement: Supplementary file 6 — Supplementary file6 (DOCX 56 KB) [file 18_2022_4137_MOESM6_ESM.docx]

Twist1 regulates macrophage plasticity to promote renal fibrosis through galectin-3

Supplementary resultS

Supplementary Figure1 The mouse model of unilateral ureteral obstruction (UUO) was successfully constructed.

Supplementary Figure 2 Macrophage-specific Twist1-deficient mice are viable and phenotypically normal.

Supplementary Figure 3 Twist1 silencing in macrophages inhibited macrophage migration.

Supplementary Figure 4 The ablation of Twist1 in macrophages has no influence on M1 macrophage polarization in the UUO and Raw264.7 cell lineage

Supplementary Figure 5 Prediction and confirmation binding site of Twist1 in the galectin-3 promotor.

Supplementary Table 1 Clinical test value of patients with IgAN.

| Histological  classification  (Lee HS) | IgAII | IgAIII | IgAIV | IgAV |
| --- | --- | --- | --- | --- |
| Age (years) | 36.3±20.6 | 31.6±20.2 | 62.9±7.7 | 58.3±8.5 |
| Sex (male/female) | 3/0 | 5/5 | 5/5 | 2/1 |
| Serum creatinine (μmol/L) | 72.33±15.50 | 72.00±10.49 | 135.90±30.52 | 186.0±11.14 |
| Serum BUN | 4.82±0.62 | 5.95±0.44 | 7.73±1.86 | 10.51±1.11 |
| eGFR (ml/min) | 105.01±20.91 | 103.10±17.1 | 47.48±8.68 | 34.75±6.62 |

Values are expressed as mean ± SD.

Supplementary Table 2 Baseline data of patients for renal biopsy samples.


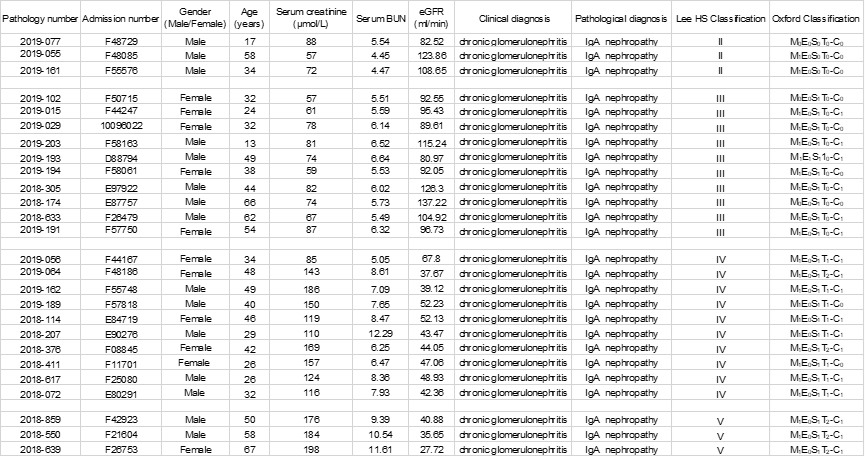


Supplementary Table 3 Real-time PCR primers.

| Gene | Primer sequence (5’ to 3’) |
| --- | --- |
| β-actin-F  β-actin-R  Twist1-F  Twist1-R  Arg1-F  Arg1-R  MR- F  MR- R  IL-10- F  IL-10- R  Fizz-F  Fizz-R  iNOS -F  iNOS- R  IL-6- F  IL-6 -R  IL1β-F  IL1β-R  CCL2-F  CCL2-R  PDGFA  PDGFA  PDGFB  PDGFB  PDGFC  PDGFC  PDGFD  PDGFD  TGFβ1  TGFβ1  TGFβ2  TGFβ2  TGFβ3  TGFβ3  VEGFA  VEGFA  CCN2  CCN2  Galectin3-F  Galectin3-R | AAACAGTCCGCCTAGAAGCAC  CGTTGACATCCGTAAAGACC  CCGGAGACCTAGATGTCATTGT  CCCACGCCCTGATTCTTGT  AGCTCTGGGAATCTGCATGG  ATGTACACGATGTCTTTGGCAGATA  AAACACAGACTGACCCTTCCC  GTTAGTGTACCGCACCCTCC  CCCTTTGCTATGGTGTCCTT  TGGTTTCTCTTCCCAAGACC  CTGCTACTGGGTGTGCTTGT  TGGTCCAGTCAACGAGTAAGC  GCAGAGATTGGAGGCCTTGTG  GGGTTGTTGCTGAACTTCCAGTC  CCACTTCACAAGTCGGAGGCTTA  GCAAGTGCATCATCGTTGTTCATAC  TCCAGGATGAGGACATGAGCAC  GAACGTCACACACCAGCAGGTTA  AGCAGCAGGTGTCCCAAAGA  GTGCTGAAGACCTTAGGGCAGA  GATGTGAGGTGAGATGAGCCG  GAGAACAAAGACCGCACGC  GCACCAACGCCAACTTCCT  GCTTCTTTCGCACAATCTCAA  CTAAAAGACCAGAGGCACCAAG  CCCATATCAAGACAACTAAGCTACA  ACAACACAGGCGGTGAAGG  GGAGAATGGAGACTAAAACGAGC  GCTAATGGTGGACCGCAAC  GCTTCCCGAATGTCTGACGTA  TCCTTCGACGTGACAGACGC  GTACAGCAGGGGCAGTGTAAAC  AAGTGGATTATCGGACGAGCC  CTTTCTAAATGACTCTGTTCACGC  CAGAAGACCGTGTGCGAATC  TGGTGGACTGAAAACTGGTATGA  GAGGAGTGGGTGTGTGACGA  GCTCGCATCATAGTTGGGTCT  CCGCTTCAATGAGAACAACAG  GGTCAGCTTCAACCAGGACTT |

Supplementary Table 4 Real-time PCR primers.

| Gene | Primer sequence (5’ to 3’) |
| --- | --- |
| Galectin-3-1MluIF  Galectin-3-1MluIR  Galectin-3-2 MluIF  Galectin-3-2 MluIR  Galectin-3-3 MluIF  Galectin-3-3 MluIR  Galectin-3-4 MluIF  Galectin-3-4 MluIR  Galectin-3-5 MluIF  Galectin-3-5 MluIR  Galectin-3-6 MluIF  Galectin-3-6 MluIR | CGACGCGTCATTCCTCTCTGCCCATCCTATTTCATG  CCGCTCGAGAGGGCGGGGCAGCATCCCTCGC  CGACGCGTATCATCTACAGATGACTCATTCAAAC  CCGCTCGAGAGGGCGGGGCAGCATCCCTCGC  CGACGCGTACAAGTGACAGCTGGAGTGCTC  CCGCTCGAGAGGGCGGGGCAGCATCCCTCGC  CGACGCGTGGGGAGAAGTCAGCAGAATGGG  CCGCTCGAGAGGGCGGGGCAGCATCCCTCGC  CGACGCGTCCCTTGCCTGAGGTAGTTGACATTG  CCGCTCGAGAGGGCGGGGCAGCATCCCTCGC  CGACGCGTTCCTCCCTTCCCTCACACTCAAAGTC  CCGCTCGAGAGGGCGGGGCAGCATCCCTCGC |

Supplementary Table 5 Real-time PCR primers.

| Gene | Primer sequence (5’ to 3’) |
| --- | --- |
| Galectin-3 ChIPF1  Galectin-3 ChIPR1  Galectin-3 ChIPF2  Galectin-3 ChIPR2  Galectin-3 ChIPF3  Galectin-3 ChIPR3  Galectin-3 distance F  Galectin-3 distance R | CCCACATGCTAATCATCTAC  GTCACCTGGCTCTCCAGATTTC  CTCTCTATACCCATATTTTC  GTCAACTACCTCAGGCAAGG  GAATCAATTCACACATTCATAC  CTAATTCCTTCACCTGAAGC  GCACAGGGATGGCCTCCATA  GTTGAACAGAGAGACAGGAC |

SUPPLEMENTARY FIGURE LEDENDS

**Supplementary Figure 1. The mouse model of unilateral ureteral obstruction (UUO) was successfully constructed. (A)** The mice were euthanized at 0, 3, 7, and 14 days after UUO, Representative images of HE, Masson, and Col1 and α-SMA immunostaining (40×) in 3-μm kidney sections. Bar scale 50 μm, n=3 samples per group, n=10 micrographs analyzed per mouse. **(B)** Bar graph analysis fibrotic area, **P* < 0.05, ***P* < 0.01. Data are presented as the mean ± SEM. Data were first analyzed for normal distribution, and if data passed normality test, 2-tailed Student’s t test for 2 groups and 2-way ANOVA for multiple groups was used.

**Supplementary Figure 2. Macrophage-specific Twist1-deficient mice are viable and phenotypically normal.** (**A**) PCR analysis for genotyping the mice. Lane 1: Lyz2-*Cre+Twist1fl/f*l; lane 2: *Lyz2-Cre-Twist1wt/fl*; and lane 3: *Lyz2-Cre-; Twist1wt/wt*. (**B**) A schematic representation of the Twist1 wild type allele and targeted locus. Ex1-2: exons 1-2. (**C**) Western blot showing Twist1 expression in macrophages pooled from 5-7 mice per group; n=3 independent experiments. (**D**) Real-time PCR of Twist1 expression in macrophages pooled from five different mice; n=3 independent experiments. (**E**) Immunostaining (40×) of Twist1 (green) and macrophage marker (red; F4/80) expression in *Cre+Twist1fl/f*l and Cre^-^Twist1^fl/fl^. Bar scale 30 μm. (**F**) Bar graph analysis of F4/80^+^Twist1^+^ macrophage numbers per high magnification view in *Cre+Twist1fl/fl* and Cre^-^Twist1^fl/fl^ mice at 14 days after unilateral ureteral obstruction (UUO), *n*=3 animals per group, *n*=10 micrographs analyzed per mouse, *Cre+Twist1fl/fl* mice versus Cre^-^Twist1^fl/fl^ mice. (**G and H**) Phenotypical analysis of *Cre+Twist1fl/fl* (n=10 animals) and Cre^-^Twist1^fl/fl^ (n=10 animals) 8-week-old mice. *Cre-Twist1fl/fl* or *Cre+Twist1fl/fl* mice showed no changes in body weight (**G**), kidney weight to body weight ratio (**H**). (**I**) Representative electron microscope and bar graph analysis of nucleus (1), mitochondria (2), basilar membrane (3) and renal tubulointerstitium (red allows) in *Cre+Twist1fl/fl* and *Cre-Twist1fl/fl* mice. Bar scale 2 μm. n=3 animals per group, n=10 micrographs analyzed per mouse, *Cre+Twist1fl/fl* and wild type littermate mice. *P < 0.05, **P < 0.01. Data are presented as the mean ± SEM. Data were first analyzed for normal distribution, and if data passed normality test, 2-tailed Student’s t test for 2 groups and 2-way ANOVA for multiple groups was used.

**Supplementary Figure 3. Twist1 deletion in macrophages inhibited macrophage migration in the unilateral ureteral obstruction (UUO) kidneys.** (**A**) Western blotting analysis Twist1 expression in Raw264.7 cell after transfection with small interfering RNA (siRNA) against Twist1. (**B**) Transwell assays of the migration and invasion abilities of Raw264.7 cells transfected with siRNA-Twist1, and the control vector (vector). (**C**) Bar graph analysis of macrophage migration number per high magnification view in Raw264.7 cell with or without downregulation of Twist1, n=10 micrographs analyzed per dish, n=3 independent experiments. (**D**) Bar graph analysis of CCL2 expression in Raw264.7 cells transfected with and without siRNA-Twist1. n=3 mice per group, n=3 independent experiments. *P < 0.05, **P < 0.01. Data are presented as the mean ± SEM. Data were first analyzed for normal distribution, and if data passed normality test, 2-tailed Student’s t test for 2 groups and 2-way ANOVA for multiple groups was used.

**Supplementary Figure 4. Ablation of Twist1 in macrophages have no influence of M1 macrophage polarization in the unilateral ureteral obstruction (UUO) kidneys and Raw264.7 cell lineage**. (**A**) Flow cytometry analysis and (**B**) bar graph analysis of M1 macrophage polarization in renal tissue of *Cre+Twist1fl/f*^l^ and wildtype littermate mice after UUO 0 day, 3 days, 7 days and 14 days, *n*=6 animals per group, *n*=3 independent experiments, *Cre+Twist1fl/f*^l^ and wild type littermate mice. (**C**) Real-time PCR analysis showing the mRNA abundance for iNOS, TNF-α, IL-6, and IL-1β in macrophages from sham and UUO kidneys at 14 days after surgery. Each sample was pooled from five individual animals within the same group. macrophages from *Cre+Twist1fl/f*^l^ versus macrophages from wildtype littermate UUO kidneys, n=3 independent experiments. (**D**) Western blotting analyses for expression of YM1 (M2 macrophage marker) in macrophages from sham and UUO kidneys at 14 days after surgery. (**E**) Real-time PCR analysis of the mRNA abundance for iNOS, TNF-α, IL-6, and IL-1β in Twist1 downregulated Raw264.7 cells, siRNA-Twist1 versus siRNA-Control (control) cells, n=3 independent experiments. Macrophages from *Cre+Twist1fl/f*^l^ versus macrophages from wild type littermate UUO kidneys, n=3 independent experiments. **P* < 0.05, ***P* < 0.01. Data are presented as the mean ± SEM. Data were first analyzed for normal distribution, and if data passed normality test, 2-tailed Student’s t test for 2 groups and 2-way ANOVA for multiple groups was used.

**Supplementary Figure 5. Twist1 binding site prediction in galectin-3 promotor.** (**A**) Twist1 binding site prediction in galectin-3 promotor in silico analysis with BiBiServ RNA hybrid. (**B**) Serially truncated Galectin-3 luciferase reporter constructs were transfected into Raw264.7 siRNA-twist1 (siTwist1) and Raw264.7 siRNA-Control (control) cells. The luciferase activity values were measured and analyzed. The luciferase values were normalized to the empty vector control. siRNA-Twist1 versus empty vector controls, n=3 independent experiments. (**C**) Diagram of the galectin-3 promotor luciferase reporter constructs containing wild-type or mutant galectin-3 binding sites. **P* < 0.05, ***P* < 0.01. Data are presented as the mean ± SEM. Data were first analyzed for normal distribution, and if data passed normality test, 2-tailed Student’s t test for 2 groups and 2-way ANOVA for multiple groups was used.
